# Synthesis and biological research of new imidazolone-sulphonamide-pyrimidine hybrids as potential EGFR-TK inhibitors and apoptosis-inducing agents†

**DOI:** 10.1039/d4ra03157a

**Published:** 2024-06-24

**Authors:** Dalal Nasser Binjawhar, Hanadi A. Katouah, Najla A. Alshaye, Jawaher Alharthi, Ghadi Alsharif, Fahmy G. Elsaid, Eman Fayad, Ali H. Abu Almaaty

**Affiliations:** a Department of Chemistry, College of Science, Princess Nourah bint Abdulrahman University P.O. Box 84428 Riyadh 11671 Saudi Arabia; b Chemistry Department, College of Science, Umm Al-Qura University 21955 Makkah Saudi Arabia; c Department of Biotechnology, College of Sciences, Taif University P.O. Box 11099 Taif 21944 Saudi Arabia; d Department of Clinical Laboratory Sciences, College of Applied Medical Sciences, King Saud Bin Abdulaziz University for Health Sciences P.O.Box 9515 Jeddah 21423 Saudi Arabia; e Department of Biomedical Research, King Abdullah International Medical Research Center 21423 Jeddah Saudi Arabia; f Department of Biology, College of Science, King Khalid University PO Box 960 Abha Asir 61421 Saudi Arabia; g Zoology Department, Faculty of Science, Port Said University Port Said 42526 Egypt aliabuelmaaty8@gmail.com

## Abstract

Development of new effective EGFR-targeted antitumor agents is needed because of their clinical significance. A new series of imidazolone-sulphonamide-pyrimidine hybrids was designed and synthesized as modified analogs of some reported EGFR inhibitors. The cytotoxic activity of all the synthesized hybrids was investigated against the breast MCF-7 cancerous cell line using doxorubicin (Dox) as a positive control. 4-(Furan-2-ylmethylene)imidazolone-sulphonamide-pyrimidine 6b had the best potent activity against MCF-7 cells with IC_50_ result of 1.05 μM, which was better than Dox (IC_50_ = 1.91 μM). In addition, mechanistic studies revealed the ability of compounds 5g, 5h and 6b to inhibit EGFR kinase. Cell cycle analysis revealed that compound 6b can halt MCF-7 cells at the G1 phase with a concomitant decrease in cellular percentage at the S and G2/M phases. This compound produced a noticeable rise in the proportion of apoptotic cells with regard to the untreated control. Furthermore, the effects of hybrid 6b on the expression levels of pro-apoptotic Bax and pro-survival Bcl2 were assessed. The results showed that this compound upregulated the level of Bax expression as well as declined the expression value of Bcl-2 with regard to the untreated control.

## Abstract 2

A sequence of novel imidazolone-sulphonamide-pyrimidine hybrids was synthesized and evaluated for *in vitro* cytotoxicity against MCF-7 breast cancer cell line.

## Graphical abstract

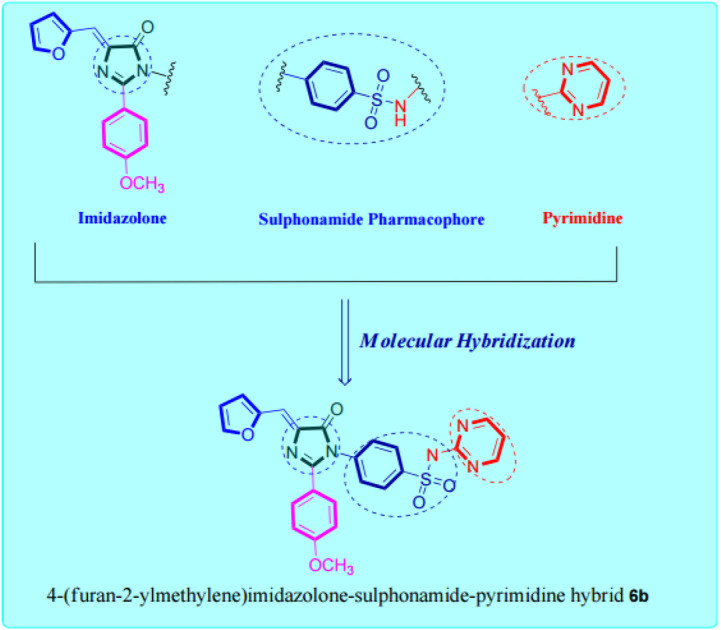

## 1. Introduction

Cancer, medically defined as a malignant neoplasm, is a class of disorders characterized by the cells dividing and growing uncontrollably forming malignant tumors, and invading neighboring parts of the body.^1,2^ Cancer can also spread *via* the lymphatic system or bloodstream to more distant areas of the body.^3^ If the spread is not controlled, it can lead to death.^4^ The development of cancer is a long-term process in which carcinogenesis events go forward step-by-step and ultimately result in the spread of cancer cells from one area of the body to other parts of the body during the metastasis stage.^5,6^ Although great improvements have been made in the survival rate from many cancers using contemporary therapeutic strategies, several side effects are unavoidable.^7^ Therefore, the search for new molecules with good antitumor effectiveness and low adverse effects is encouraging.^8^ Molecular breakthroughs have led to the discovery of cellular parts involved in the initiation, propagation and proliferation of lesions that lead to primary cancer and metastases.^9–11^ These may be an attractive proposition for cancer treatment.^12^ Thus, focusing on these biological targets that may inhibit or reverse the interrelated processes is crucial for cancer remedy.^13,14^ Kinases have emerged as one of the most powerful intensive classes of therapeutic targets, with several kinase targets being explored to the stage of clinical trials.^15,16^ Epidermal growth factor receptor (EGFR) represents one of the most significant validated cancer therapeutics.^17^ In recent years, a substantial number of small molecule EGFR-kinase inhibitors with high antiproliferative activity have been described.^18,19^ The EGFR inhibitors have some essential pharmacophoric features that can occupy a specific pocket in the ATP binding site of EGFR.^20^ The pharmacophoric features include a heterocyclic system that is typically supplemented by two hydrophobic groups, a small spacer group, hydrogen bond donor–acceptor group, and finally flat heterocyclic ring connected to the allosteric site outside the ATP binding site.^21^ An alternative chemotherapeutic strategy involves the use of agents that can induce cellular apoptosis to rapidly eradicate premalignant instead of just inhibiting their growth and/or encouraging some degree of differentiation.^22,23^ Indeed, activation of apoptosis is becoming more valued as a biologically meaningful anticancer method in the realm of cancer therapy.^24^

**Fig. 1:**
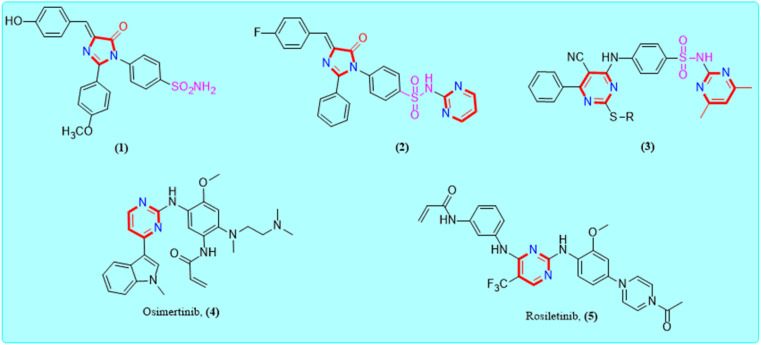
Examples of some reported imidazolone, sulphonamide and clinically approved pyrimidine scaffold as anti-EGFR candidates.

The imidazolone framework is a remarkable heterocyclic motif that has a unique function in the development of anti-cancer drugs.^25,26^ So far, a variety of imidazolones with different substitution patterns have been developed and validated against numerous molecular targets for the treatment of cancer molecules with outstanding outcomes.^27^ The imidazolone pharmacophore can acquire synergistic activity with other agents and also, re-sensitize that have acquired resistance.^28^ Alternatively, sulphonamide and its derivatives are bestowed with diverse biological properties and have received significant attention in the drug development process.^29,30^ Moreover, from a chemical structure point of view, the common skeleton of several kinase inhibitors is the pyrimidine ring^31^ (Fig. 1).

In this investigation, a new set of imidazolone-sulphonamide-pyrimidine hybrids 5a–l and 6a,b were designed and synthesized. The cytotoxic activity of the new imidazolone-sulphonamide-pyrimidine hybrids was assessed on the breast MCF-7 cancer cell line. In addition, the most potent hybrid molecules were evaluated *in vitro* to assess their EGFR inhibitory activities. Furthermore, a flow cytometric examination of the most active member was also carried out in the study in order to ascertain if the cytotoxic effect is accompanied by a shift in the cell cycle. Moreover, the ability to downregulate Bcl-2 and activate Bax was examined in order to evaluate apoptotic induction and reveal the mechanism of how the active hybrid killed MCF-7 cells.

## 2. Results and discussion

### 2.1. Chemistry

**Scheme 1:**
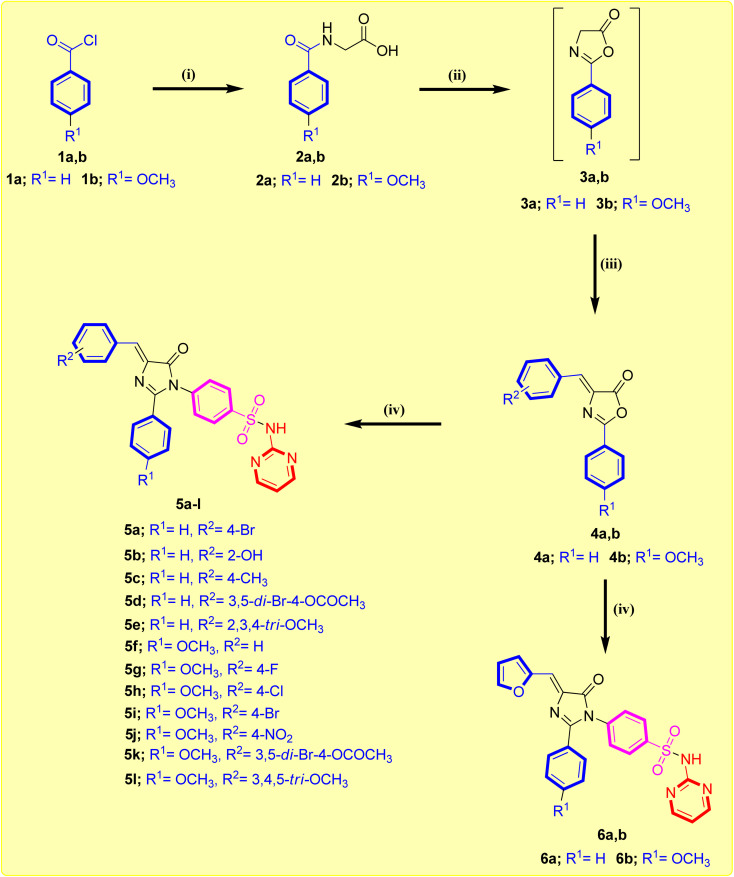
Synthesis of imidazolone-sulphonamide-pyrimidine compounds 5a–l and 6a,b. Reagents: (i) glycine, aqueous sodium hydroxide, rt, 2 h; (ii) Ac_2_O, 80 °C, 2 h; (iii) respective aryl aldehyde, NaOAc, 80 °C, 2 h; (iv) sulfadiazine, NaOAc, glacial acetic acid, reflux 16–18 h.

The synthetic procedure adopted to prepare the newly synthesized imidazolone-sulphonamide-pyrimidine hybrids 5a–l and 6a,b is illustrated in Scheme 1. In the initial step appropriate hippuric acid 2a,b was obtained by stirring appropriate aroyl chloride 1a,b and glycine in an aqueous sodium hydroxide solution, which was then heated in acetic anhydride at 80 °C to obtain active oxazolone compound 3a,b.^32^ This was followed by condensation with suitable aryl aldehyde in the presence of anhydrous pyridine furnished the key intermediate 4-arylidene oxazolone compound 4a,b.^33^ Refluxing acetic acid suspension of appropriate 4-arylidene oxazolone compound 4a,b with sulfadiazine in the existence of freshly melted sodium acetate afforded the titled compound 5a–l and 6a,b. The yields of the title molecules ranged from 53–78% after recrystallization with aqueous ethanol (70%). The purity of the title molecules was reviewed by TLC utilizing *n*-hexane/ethyl acetate (1 : 3) as the eluent and elemental analyses. Both the analytical and spectral results (^1^H-NMR and ^13^C-NMR) of all the synthesized hybrids were completely consistent with the speculated structures.

In the nuclear magnetic resonance spectra (^1^H-NMR) the signals of the particular protons of the prepared title were attained by employing their chemical shifts, multiplicities and coupling constants. Compounds 5f-5e and 6b showed a singlet peak in the range of *δ* 3.78–3.85 ppm related to the methoxy (OCH_3_) group in the benzene ring. ^1^H-NMR spectra of imidazolone compounds 5a–l and 6a,b showed a common signal that appeared as a broad singlet between *δ* 11.20–12.07 ppm attributed to sulphamoyl –SO_2_NH proton. In addition, common signals appeared as a doublet in the range of *δ* 8.42–8.54 ppm corresponding to C3,5-Hs of the 1,3-diazine ring; triplet at *δ* 6.93–7.10 ppm related C4–H of the 1,3-diazine ring; singlet at *δ* 7.00–7.32 ppm corresponding to olefinic (CH=) hydrogen. The elemental analysis findings were around ±0.4% of the recommended value. On the other hand, ^13^C-NMR spectra of the compounds 5f-5e and 6b showed a common carbon signal appearing at *δ* 55.88–56.08 ppm related to the methoxy (OCH_3_) carbon of the benzene ring. In ^13^C-NMR spectra of compounds 5a–6b, peaks of olefinic and aromatic carbons were found at *δ* 109.28–162.48 ppm. Moreover, the ^13^C-NMR spectra of imidazolone derivatives 5a–6b showed a common characteristic signal at *δ* 157.20–158.65 ppm attributed to C2 of the imidazolone ring and another peak related C=O of the imidazolone ring in the range at *δ* 169.22–169.82 ppm. In addition, two new signals at *δ* 158.31–161.84 and 159.53–164.80 ppm were attributed to C3,5 and C1 of the 1,3-diazine moiety. Besides, ^1^H-NMR and ^13^C-NMR spectra of all imidazolone-sulphonamide-pyrimidine hybrids 5a–l and 6a,b are presented in the ESI.†

### 2.2. Biology

#### 2.2.1. Assessment of cytotoxic effectiveness

The MTT colorimetric method, a frequently utilized assay for detecting the *in vitro* cytotoxic activities of drugs, was chosen to evaluate the cytotoxic action of the imidazolone-sulphonamide-pyrimidine hybrids 5a–l and 6a,b in hands against breast MCF-7 adenocarcinoma cell line, and they were compared to Dox as the positive control. The obtained data are presented in Table 1. The results demonstrated that the tested imidazolone-sulphonamide-pyrimidine molecules showed significant cytotoxic activity against the tested MCF-7 cells and their potency was affected by both the nature and the type of substituents on the C2-aryl and C4-arylidene moieties of the imidazolone function. The imidazolone compound 6b bearing heterocyclic ring-like 4-(furan-2-yl)methylene group was the most potent hybrid molecule in this study (IC_50_ = 1.05 μM) compared with Dox (IC_50_ = 1.91 μM). In addition, the potency was also favored by grafting an electron-withdrawing substituent to benzylidene function on the imidazolone ring-like chlorine; 5h (IC_50_ = 3.71 μM) or nitro; 5j (IC_50_ = 3.14 μM). On the other hand, introducing electron-donating substituent on the benzylidene ring as methyl or methoxy group resulted in compounds having moderate cytotoxic activity. Moreover, changing the C2-phenyl imidazolone to C2-(4-methoxyphenyl) imidazolone resulted in compounds having good cytotoxic activity with IC_50_ values between 1.05 to 25.79 μM.

**Table 1:** IC_50_ values (μM) of tested 4-(arylidene)imidazolone-sulphonamide-pyrimidine hybrids 5a–l and 6a,b against breast MCF-7 cancerous cell line

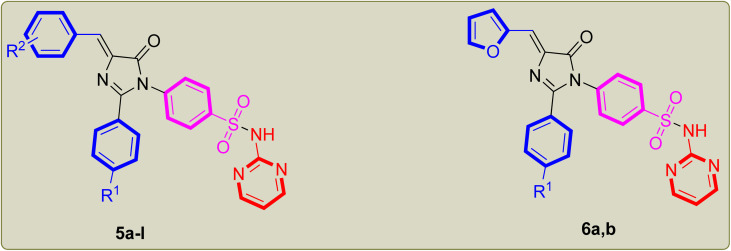			
Comp. No.	R^1^	R^2^	IC_50_ (μM)
			MCF-7
5a	H	4-Br	14.05 ± 0.68
5b	H	2-OH	31.44 ± 1.64
5c	H	4-CH_3_	43.94 ± 2.24
5d	H	3,5-di-Br-4-OCOCH_3_	29.35 ± 1.64
5e	H	2,3,4-tri-OCH_3_	37.23 ± 1.98
5f	OCH_3_	H	85.23 ± 4.72
5g	OCH_3_	4-F	4.57 ± 0.09
5h	OCH_3_	4-Cl	3.71 ± 0.20
5i	OCH_3_	4-Br	9.98 ± 0.61
5j	OCH_3_	4-NO_2_	3.14 ± 0.16
5k	OCH_3_	3,5-di-Br-4-OCOCH_3_	14.46 ± 0.65
5l	OCH_3_	3,4,5-tri-OCH_3_	25.79 ± 1.24
6a	H	—	5.09 ± 0.31
6b	OCH_3_	—	1.05 ± 0.04
Dox	—	—	1.91 ± 0.07

#### 2.2.2. *In vitro* EGFR kinase inhibitory activity

**Fig. 2:**
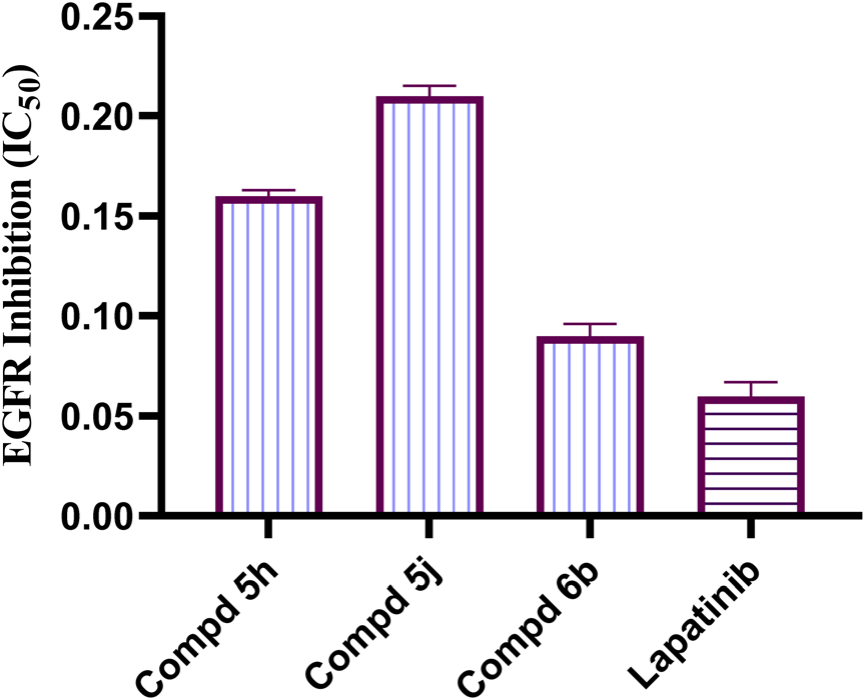
Graphical illustration of IC_50_ (μM) for imidazolone-sulphonamide-pyrimidine hybrids 5h, 5j, 6b and Lapatinib on EGFR enzyme. Values indicate the mean ± SE for three replicates.

EGFR-TK is a well-known and validated anticancer target with several clinically used drugs.^34^ To examine the potential mechanism falling behind the high anticancer effects of imidazolone-sulphonamide-pyrimidine hybrids, the inhibitory potential was evaluated against EGFR-TK. The most effective cytotoxic hybrid molecules 5h, 5j and 6b were assessed for their suppressive efficacy against EGFR-TK. The results were given as an IC_50_ value calculated from the concentration inhibition responsiveness graph, and they are presented in Fig. 2. Lapatinib as a reference EGFR inhibitor was used and also tested as the positive control during this experiment. The tested imidazolone hybrids demonstrated high to adequate inhibitory efficacy with an IC_50_ value fluctuating between 0.09–0.21 μM. Among them, 4-(furan-2-ylmethylene)imidazolone-sulphonamide-pyrimidine 6b came out to be the most effective derivative inhibiting EGFR at an IC_50_ value of 0.09 μM, this is nearly the same as that of standard drug Lapatinib with an IC_50_ result of 0.06 μM. Furthermore, both 4-(4-chlorobenzylidene)imidazolone-sulphonamide-pyrimidine 5h and 4-(4-nitrobenzylidene)imidazolone-sulphonamide-pyrimidine 5j hybrids possessed a very good EGFR inhibition with an IC_50_ results of 0.16 and 0.21 μM, respectively.

#### 2.2.3. Cell cycle analysis

**Fig. 3:**
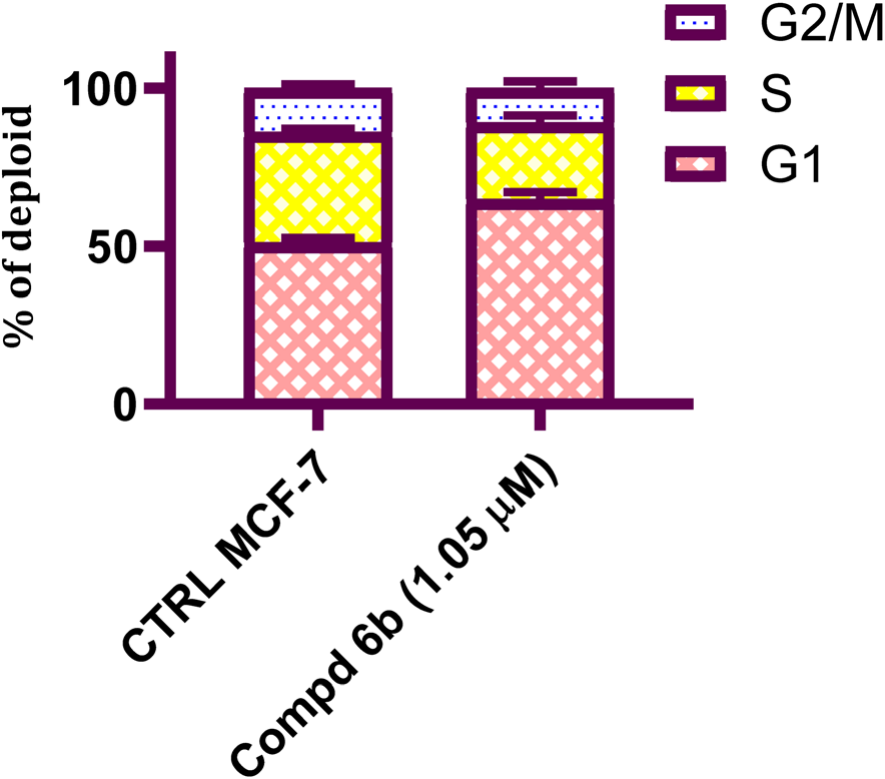
Cell cycle investigation of phosphatidylserine externalized Annexin-V interaction and cell membrane integrity (PI labelling). MCF-7 cells underwent treatment with 4-(furan-2-ylmethylene)imidazolone-sulphonamide-pyrimidine 6b at (A) 0, (B) 1.05 μM, respectively.

The impact of the best potent hybrid molecule on the cellular cycle distribution was further evaluated on the MCF-7 cell line. The tested cell line received treatment with 4-(furan-2-ylmethylene)imidazolone-sulphonamide-pyrimidine 6b with a dose of 1.05 μM (IC_50_ result) over 48 h. The results revealed that 6b caused a significant accumulation of MCF-7 cells in the G1 phase (64.9%) at the IC_50_ concentration as compared with the control (51.2%). While, a reduced accumulation was observed in the S phase (24.3%) and G2/M phase (10.8%) as compared to the control, which showed 35.1% accumulation in the S phase and 13.7%, in the S phase. These overall results suggested that 4-(furan-2-ylmethylene)imidazolone-sulphonamide-pyrimidine hybrid 6b halts the proliferation of MCF-7 cells by arresting them at the G1 phase, which ultimately leads to cellular death (Fig. 3).

#### 2.2.4. Apoptosis staining assay

**Fig. 4:**
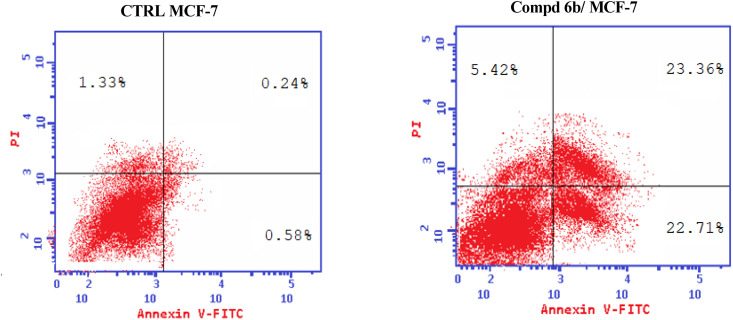
Flow cytometric method by Annexin V/PI method of the tested MCF-7 cells treated with 4-(furan-2-ylmethylene)imidazolone-sulphonamide-pyrimidine hybrid 6b at the IC_50_ compared with having no treatment control for 48 h.

Apoptosis, a form of programmed cellular death, is a widespread phenomenon, occurring in processes such as morphogenesis, growth and development as well as normal turnover in adult tissue.^35^ Most cells are programmed to die if survival signals are not received regularly from their environment.^36^ It can be initiated by endogenous tissue-specific agents such as cytokines or exogenous cell-damaging agents such as chemicals.^37^ It is evidenced from the literature that exposure to EGFR-TK inhibitors leads to cellular apoptosis.^38^ Thus, the effect of examined 4-(furan-2-ylmethylene)imidazolone-sulphonamide-pyrimidine hybrid 6b on the apoptosis of the MCF-7 cells was assessed utilizing Annexin V-FITC/PI double labeling followed by flow cytometry (Fig. 4). Annexin V-FITC/PI flow cytometry analysis revealed that MCF-7 cells undergo apoptosis after treatment with IC_50_ concentration of the compound 6b as shown in Fig. 4. 4-(furan-2-ylmethylene)imidazolone-sulphonamide-pyrimidine hybrid 6b induced 46.1% apoptosis in the MCF-7 cancer cells. Thus, it is evident that this compound had substantial apoptosis-inducing action against the MCF-7 breast cancerous cell line.

#### 2.2.5. Compound 6b regulates the expression of the Bcl-2 family of proteins

**Fig. 5:**
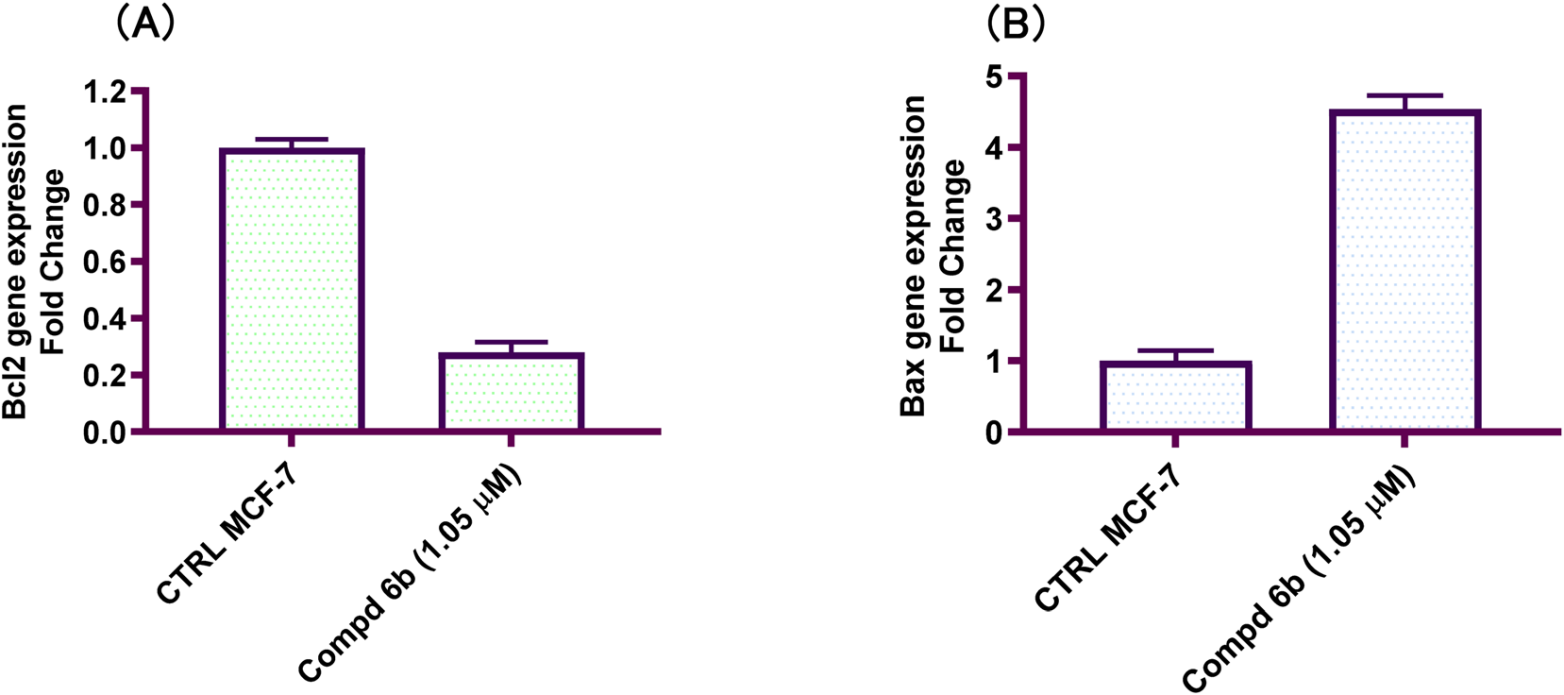
Roles of Bcl-2 family protein in apoptosis induced by 4-(furan-2-ylmethylene)imidazolone-sulphonamide-pyrimidine hybrid 6b. (A) Pro-survival Bcl-2 protein; (B) pro-apoptotic Bax protein. Cells underwent treatment with an IC_50_ concentration of imidazolone hybrids 6b for 48 h and the expression of apoptosis-related proteins was examined by qRT-PCR assay.

It is also evidenced from the literature that a link between apoptosis and mitochondrial physiology is suggested by the presence of the Bcl-2 family of proteins in the mitochondrial membrane.^39^ The Bcl-2 family members are essential in determining whether a cell will survive or perish since they reside upstream of the irreversible cellular damage.^40^ Two groups of the Bcl-2 family proteins are distinguished by pro-survival (*e.g.* Bcl-2) or pro-apoptotic (Bax) activity.^41^ The pro-apoptotic factors promote cellular death. When Bcl-2-associated x-protein (Bax) homodimerizes it enhances apoptosis. The anti-apoptotic effect of Bcl-2 may be due to its ability to inhibit Bax homodimerization.^42,43^ In this study, the findings of qRT-PCR revealed that 4-(furan-2-ylmethylene)imidazolone-sulphonamide-pyrimidine hybrid 6b inhibited the expression of pro-survival Bcl-2, and upregulated the expression of pro-apoptotic Bax. It is noteworthy, that the level of pro-survival Bcl-2 protein declined by 0.28-fold compared to the control untreated cells. On the other hand, the level of the pro-apoptotic Bax was increased by 4.54-fold as compared with the untreated control (Fig. 5). These findings support that 4-(furan-2-ylmethylene)imidazolone-sulphonamide-pyrimidine hybrid 6b induces apoptosis by regulating the expression of Bcl-2 family proteins.

## 3. Conclusions

In this study, a congeneric series of imidazolone-sulphonamide-pyrimidine hybrids 5a–l and 6a,b were designed and synthesized as potential anticancer agents. The results indicated that 4-(4-chlorobenzylidene)imidazolone-sulphonamide-pyrimidine hybrid 5h (IC_50_ = 3.71 μM), 4-(4-nitrobenzylidene)imidazolone-sulphonamide-pyrimidine hybrid 5j (IC_50_ = 3.14 μM) and 4-(furan-2-ylmethylene)imidazolone-sulphonamide-pyrimidine hybrid 6b (IC_50_ = 1.05 μM) were the most effective hybrids against the MCF-7 cell line compared to the Dox as positive control (IC_50_ = 1.91 μM). In addition, 4-(furan-2-ylmethylene)imidazolone-sulphonamide-pyrimidine hybrid 6b showed promising EGFR inhibitory activity (IC_50_ = 0.09 μM) compared to Lapatinib (IC_50_ = 0.06 μM). Additionally, 4-(4-chlorobenzylidene)imidazolone-sulphonamide-pyrimidine 5h (IC_50_ = 0.16 μM) and 4-(4-nitrobenzylidene)imidazolone-sulphonamide-pyrimidine 5j (IC_50_ = 0.21 μM) showed good EGFR inhibitory activity. Furthermore, 4-(furan-2-ylmethylene)imidazolone-sulphonamide-pyrimidine hybrid 6b exerted a notable rise in the population of MCF-7 cells at the G1 phase from 51.2 to 64.9%. Moreover, 4-(furan-2-ylmethylene)imidazolone-sulphonamide-pyrimidine hybrid 6b exerted a significant increase in the apoptotic cells in the early stage from 0.6 to 22.7%, and in the late stage from 0.2 to 23.4%. Finally, 4-(furan-2-ylmethylene)imidazolone hybrid 6b produced a significant increase in the expression value of Bcl-2 associated x-protein (Bax) by 4.54-fold. On the other hand, this compound caused a significant decrease in the expression value of the pro-survival marker Bcl-2 by 0.28-fold with regard to the no-treatment control.

## 4. Experimental

### 4.1. Chemistry

#### 4.1.1 The general method for the preparation of (*Z*)-4-(4-arylidene)-5-oxo-2-aryl-4,5-dihydro-1*H*-imidazole-1-yl-*N*-(pyrimidin-2-yl)benzenesulfonamides 5a–l and 6a,b

To a suspension of the respective oxazolone (0.12 mmol) in glacial acetic acid (20 mL), 4-amino-*N*-(pyrimidin-2-yl)benezenesulphonamide (3.50 g, 0.14 mmol) was placed. The reaction mixture was refluxed for 16–18 h. Once the reaction was completed (consumption of oxazolone molecule), the resulting mixture was kept refrigerated for one night. The precipitated residue was gathered by using filtration and rinsed with absolute ethanol (10 mL). The product solid was purified from aqueous ethanol by crystallization to attain imidazolone-sulphonamide-pyrimidine hybrid 5a–l and 6a,b.

##### 4.1.1.1 (*Z*)-4-(4-(4-bromobenzylidene)-5-oxo-2-phenyl-4,5-dihydro-1*H*-imidazole-1-yl)-*N*-(pyrimidin-2-yl)benzenesulfonamide (5a)

Yield: 74%, m.p. 290–292 °C. ^1^H-NMR (DMSo-*d*_6_, 400 MHz) *δ* (ppm): 11.91 (s, 1H, NH), 8.51 (d, *J* = 4.9 Hz, 2H, C3,5-H pyrimidine), 8.31 (d, *J* = 8.6 Hz, 2H, Ar–H), 8.03 (d, *J* = 8.6 Hz, 2H, Ar–H), 7.73 (d, *J* = 8.6 Hz, 2H, Ar–H), 7.52 (t, *J* = 7.3 Hz, 1H, Ar–H), 7.49–7.46 (m, 2H, Ar–H), 7.46–7.44 (m, 2H, Ar–H), 7.37 (t, *J* = 7.7 Hz, 2H, Ar–H), 7.31 (s, 1H, CH=N), 7.07 (t, *J* = 4.9 Hz, 1H, C4–H pyrimidine). ^13^C-NMR (DMSo, 101 MHz) *δ*: 169.55, 161.19, 158.74, 157.56, 140.92, 139.17, 138.24, 134.58, 134.49, 133.68, 132.43, 132.20, 129.45, 128.99, 128.68, 128.44, 127.14, 127.05, 124.84. Analysis: calc. for C_26_H_18_BrN_5_O_3_S (560.42): C 55.72, H 3.24, N 12.50%, found: C 55.81, H 3.29, N 12.39%.

##### 4.1.1.2 (*Z*)-4-(4-(2-hydroxybenzylidene)-5-oxo-2-phenyl-4,5-dihydro-1*H*-imidazole-1-yl)-*N*-(pyrimidin-2-yl)benzenesulfonamide (5b)

Yield: 53%, m.p. 188–190 °C. ^1^H-NMR (DMSo-*d*_6_, 400 MHz) *δ* (ppm): 11.20 (s, 1H, NH), 9.67 (s, 1H, OH), 8.64–8.48 (m, 1H, C3,5-H pyrimidine), 8.53–8.45 (m, 1H, Ar–H), 8.02 (d, *J* = 7.0 Hz, 1H, Ar–H), 7.98 (d, *J* = 7.5 Hz, 2H, Ar–H), 7.79 (d, *J* = 7.5 Hz, 1H, Ar–H), 7.65 (t, *J* = 7.3 Hz, 1H, Ar–H), 7.60 (d, *J* = 8.4 Hz, 1H, Ar–H), 7.58–7.49 (m, 3H, Ar–H), 7.46 (d, *J* = 8.2 Hz, 2H, Ar–H), 7.42 (d, *J* = 5.9 Hz, 1H, Ar–H), 7.40–7.27 (m, 2H, Ar–H and CH=N), 7.06–6.93 (m, 1H, C4–H pyrimidine). ^13^C-NMR (DMSo, 101 MHz) *δ*: 166.37, 158.58, 158.31, 150.67, 133.92, 132.77, 130.74, 129.49, 129.15, 128.96, 128.92, 128.62, 128.37, 128.11, 128.04, 127.45, 127.34, 125.53, 124.62, 119.79, 116.46. Analysis: calc. for C_26_H_19_N_5_O_4_S (497.53): C 62.77, H 3.85, N 14.08%, found: C 62.94, H 3.76, N 13.96%.

##### 4.1.1.3 (*Z*)-4-(4-(4-methylbenzylidene)-5-oxo-2-phenyl-4,5-dihydro-1*H*-imidazole-1-yl)-*N*-(pyrimidin-2-yl)benzenesulfonamide (5c)

Yield: 68%, m.p. 277–279 °C. ^1^H-NMR (DMSo-*d*_6_, 400 MHz) *δ* (ppm): 11.98 (s, 1H, NH), 8.52 (d, *J* = 4.9 Hz, 2H, C3,5-H pyrimidine), 8.26 (d, *J* = 8.2 Hz, 2H, Ar–H), 8.03 (d, *J* = 8.5 Hz, 2H, Ar–H), 7.51 (t, *J* = 7.4 Hz, 1H, Ar–H), 7.48 (s, 1H, Ar–H), 7.46 (s, 1H, Ar–H), 7.46–7.43 (m, 2H, Ar–H), 7.38 (d, *J* = 7.6 Hz, 2H, Ar–H), 7.34 (d, *J* = 7.7 Hz, 2H, Ar–H), 7.29 (s, 1H, CH=N), 7.09 (t, *J* = 4.9 Hz, 1H, C4–H pyrimidine), 2.39 (s, 3H, CH_3_). ^13^C-NMR (DMSo, 101 MHz) *δ*: 169.60, 160.11, 158.73, 157.30, 141.55, 138.50, 137.91, 132.99, 132.88, 131.97, 131.77, 130.11, 130.04, 129.36, 128.96, 128.94, 128.90, 128.85, 128.44, 21.71. Analysis: calc. for C_27_H_21_N_5_O_3_S (495.55): C 65.44, H 4.27, N 14.13%, found: C 65.35, H 4.38, N 14.23%.

##### 4.1.1.4 (*Z*)-2,6-dibromo-4-((5-oxo-2-phenyl-1-(4-(*N*-pyrimidin-2-ylsulfamoyl)phenyl)-1*H*-imidazole-4(5*H*)-ylidene)methyl)phenyl acetate (5d)

Yield: 72%, m.p. 243–245 °C. ^1^H-NMR (DMSo-*d*_6_, 400 MHz) *δ* (ppm): 8.76 (s, 2H, Ar–H), 8.42 (d, *J* = 4.8 Hz, 2H, C3,5-H pyrimidine), 7.99 (d, *J* = 8.4 Hz, 2H, Ar–H), 7.54 (t, *J* = 7.3 Hz, 1H, Ar–H), 7.47 (s, 1H, Ar–H), 7.45 (d, *J* = 5.3 Hz, 2H, Ar–H), 7.41 (d, *J* = 8.4 Hz, 2H, Ar–H), 7.38–7.34 (m, 1H, Ar–H), 7.31 (s, 1H, CH=N), 6.93 (t, *J* = 4.7 Hz, 1H, C4–H pyrimidine), 2.44 (s, 3H, CH_3_). ^13^C-NMR (DMSO, 101 MHz) *δ*: 172.48, 169.41, 167.66, 162.48, 158.48, 146.80, 140.40, 135.88, 135.60, 132.47, 129.46, 129.02, 128.83, 128.72, 128.50, 128.23, 128.06, 123.90, 117.96, 20.69. Analysis: calc. for C_28_H_19_Br_2_N_5_O_5_S (697.35): C 48.23, H 2.75, N 10.04%, found: C 48.36, H 2.83, N 9.95%.

##### 4.1.1.5 (*Z*)-4-(5-oxo-2-phenyl-4-(2,3,4-trimethoxybenzylidene)-4,5-dihydro-1*H*-imidazole-1-yl)-*N*-(pyrimidin-2-yl)benzenesulfonamide (5e)

Yield: 57%, m.p. 210–212 °C. ^1^H-NMR (DMSo-*d*_6_, 400 MHz) *δ* (ppm): 11.98 (s, 1H, NH), 8.73 (d, *J* = 9.0 Hz, 1H, Ar–H), 8.53 (d, *J* = 4.9 Hz, 2H, C3,5-H pyrimidine), 8.03 (d, *J* = 8.5 Hz, 2H, Ar–H), 7.51 (d, *J* = 7.2 Hz, 1H, Ar–H), 7.47 (s, 1H, Ar–H), 7.45 (s, 1H, Ar–H), 7.44 (s, 2H, Ar–H), 7.43 (s, 1H, CH=N), 7.36 (t, *J* = 7.6 Hz, 2H, Ar–H), 7.10 (t, *J* = 4.9 Hz, 1H, C4-H pyrimidine), 7.06 (d, *J* = 9.1 Hz, 1H, Ar–H), 3.92 (s, 3H, OCH_3_), 3.90 (s, 3H, OCH_3_), 3.80 (s, 3H, OCH_3_). ^13^C-NMR (DMSo, 101 MHz) *δ*: 169.58, 159.53, 158.76, 158.69, 157.21, 156.68, 154.31, 152.98, 141.87, 140.44, 138.60, 137.15, 131.91, 129.30, 128.98, 128.90, 128.60, 128.45, 122.11, 120.76, 109.28, 62.41, 60.99, 56.60. Analysis: calc. for C_29_H_25_N_5_O_6_S (571.60): C 60.94, H 4.41, N 12.25%, found: C 61.08, H 4.53, N 12.13%.

##### 4.1.1.6 (*Z*)-4-(4-benzylidene-2-(4-methoxyphenyl)-5-oxo-4,5-dihydro-1*H*-imidazole-1-yl)-*N*-(pyrimidin-2-yl)benzenesulfonamide (5f)

Yield: 64%, m.p. 258–260 °C. ^1^H-NMR (DMSo-*d*_6_, 400 MHz) *δ* (ppm): 11.97 (s, 1H, NH), 8.53 (d, *J* = 4.9 Hz, 2H, C3,5-H pyrimidine), 8.36 (d, *J* = 8.8 Hz, 2H, Ar–H), 8.03 (d, *J* = 8.5 Hz, 2H, Ar–H), 7.51 (d, *J* = 7.3 Hz, 1H, Ar–H), 7.46 (d, *J* = 8.3 Hz, 3H, Ar–H), 7.44 (s, 1H, Ar–H), 7.36 (t, *J* = 7.7 Hz, 2H, Ar–H), 7.29 (s, 1H, CH=N), 7.11 (d, *J* = 4.2 Hz, 2H, Ar–H), 7.09 (s, 1H, C4-H pyrimidine), 3.85 (s, 3H, OCH_3_). ^13^C-NMR (DMSo, 101 MHz) *δ*: 169.53, 161.94, 159.21, 158.81, 157.20, 140.41, 138.65, 136.60, 135.02, 131.83, 129.29, 129.06, 128.97, 128.94, 128.89, 128.45, 127.23, 115.07, 55.92. Analysis: calc. for C_27_H_21_N_5_O_4_S (511.55): C 63.39, H 4.14, N 13.69%, found: C 63.29, H 4.02, N 13.76%.

##### 4.1.1.7 (*Z*)-4-(4-(4-fluorobenzylidene)-2-(4-methoxyphenyl)-5-oxo-4,5- dihydro-1*H*-imidazole-1-yl)-*N*-(pyrimidin-2-yl)benzenesulfonamide (5g)

Yield: 69%, m.p. 257–259 °C. ^1^H-NMR (DMSo-*d*_6_, 400 MHz) *δ* (ppm): 12.04 (s, 1H, NH), 8.54 (d, *J* = 4.8 Hz, 2H, C3,5-H pyrimidine), 8.49–8.40 (m, 2H, Ar–H), 8.06 (d, *J* = 8.3 Hz, 2H, Ar–H), 7.50 (d, *J* = 8.3 Hz, 2H, Ar–H), 7.42 (d, *J* = 8.7 Hz, 2H, Ar–H), 7.37 (t, *J* = 8.8 Hz, 2H, Ar–H), 7.26 (s, 1H, CH=N), 7.10 (t, *J* = 4.7 Hz, 1H, C4-H pyrimidine), 6.91 (d, *J* = 8.7 Hz, 2H, Ar–H), 3.79 (s, 3H, OCH_3_). ^13^C-NMR (DMSo, 101 MHz) *δ*: 172.48, 169.79, 164.80, 162.39, 160.28, 157.24, 140.62, 138.74, 138.36, 135.10, 131.34, 131.27, 129.02, 128.61, 126.15, 126.02, 120.65, 116.64, 116.36, 114.54, 114.39, 55.88. Analysis: calc. for C_27_H_20_FN_5_O_4_S (529.54): C 61.24, H 3.81, N 13.23%, found: C 61.11, H 3.89, N 13.32%.

##### 4.1.1.8 (*Z*)-4-(4-(4-chlorobenzylidene)-2-(4-methoxyphenyl)-5-oxo-4,5-dihydro-1*H*-imidazole-1-yl)-*N*-(pyrimidin-2-yl)benzenesulfonamide (5h)

Yield: 74%, m.p. 262–264 °C. ^1^H-NMR (DMSo-*d*_6_, 400 MHz) *δ* (ppm): 12.07 (s, 1H, NH), 8.54 (d, *J* = 4.9 Hz, 2H, C3,5-H pyrimidine), 8.38 (d, *J* = 8.6 Hz, 2H, Ar–H), 8.10–8.04 (m, 2H, Ar–H), 7.60 (t, *J* = 8.0 Hz, 2H, Ar–H), 7.50 (d, *J* = 8.5 Hz, 2H, Ar–H), 7.42 (d, *J* = 8.9 Hz, 2H, Ar–H), 7.24 (s, 1H, CH=N), 7.10 (t, *J* = 4.8 Hz, 1H, C4-H pyrimidine), 6.92 (d, *J* = 8.9 Hz, 2H, Ar–H), 3.79 (s, 3H, OCH_3_). ^13^C-NMR (DMSo, 101 MHz) *δ*: 169.74, 162.47, 160.64, 139.16, 135.45, 134.30, 134.15, 133.57, 132.96, 131.40, 131.34, 129.54, 129.45, 129.03, 128.64, 125.63, 120.57, 114.55, 114.41, 56.07. Analysis: calc. for C_27_H_20_ClN_5_O_4_S (546.00): C 59.39, H 3.69, N 12.83%, found: C 59.55, H 3.81, N 12.68%.

##### 4.1.1.9 (*Z*)-4-(4-(4-bromobenzylidene)-2-(4-methoxyphenyl)-5-oxo-4,5-dihydro-1*H*-imidazole-1-yl)-*N*-(pyrimidin-2-yl)benzenesulfonamide (5i)

Yield: 78%, m.p. 284–286 °C. ^1^H-NMR (DMSo-*d*_6_, 400 MHz) *δ* (ppm): 8.50 (d, *J* = 4.9 Hz, 2H, C3,5-H pyrimidine), 8.30 (d, *J* = 8.6 Hz, 2H, Ar–H), 8.05 (d, *J* = 8.5 Hz, 2H, Ar–H), 7.73 (d, *J* = 8.5 Hz, 2H, Ar–H), 7.48 (d, *J* = 8.5 Hz, 2H, Ar–H), 7.42 (d, *J* = 8.8 Hz, 2H, Ar–H), 7.22 (s, 1H, CH=N), 7.04 (t, *J* = 4.8 Hz, 1H, C4-H pyrimidine), 6.92 (d, *J* = 8.9 Hz, 2H, Ar–H), 3.79 (s, 3H, OCH_3_). ^13^C-NMR (DMSo, 101 MHz) *δ*: 169.78, 162.48, 160.70, 158.65, 139.27, 138.43, 134.43, 134.31, 133.88, 132.38, 131.41, 131.35, 129.07, 128.95, 128.54, 125.78, 125.67, 124.49, 120.58, 114.56, 114.40, 56.08. Analysis: calc. for C_27_H_20_BrN_5_O_4_S (590.45): C 54.92, H 3.41, N 11.86%, found: C 55.10, H 3.58, N 11.67%.

##### 4.1.1.10 (*Z*)-4-(2-(4-methoxyphenyl)-4-(4-nitrobenzylidene)-5-oxo-4,5-dihydro-1*H*-imidazole-1-yl)-*N*-(pyrimidin-2-yl)benzenesulfonamide (5j)

Yield: 75%, m.p. > 300 °C. ^1^H-NMR (DMSo-*d*_6_, 400 MHz) *δ* (ppm): 8.59 (d, *J* = 9.0 Hz, 2H, Ar–H), 8.53 (d, *J* = 4.9 Hz, 2H, C3,5-H pyrimidine), 8.34 (d, *J* = 9.0 Hz, 2H, Ar–H), 8.12–8.04 (m, 2H, Ar–H), 7.52 (d, *J* = 8.6 Hz, 2H, Ar–H), 7.46 (d, *J* = 8.9 Hz, 2H, Ar–H), 7.32 (s, 1H, CH=N), 7.07 (t, *J* = 4.9 Hz, 1H, C4-H pyrimidine), 6.93 (d, *J* = 9.0 Hz, 2H, Ar–H), 3.80 (s, 3H, OCH_3_). ^13^C-NMR (DMSo, 101 MHz) *δ*: 169.78, 162.82, 162.41, 158.83, 157.53, 147.78, 141.42, 141.10, 138.43, 133.33, 133.25, 131.64, 129.16, 128.65, 124.35, 124.23, 123.63, 123.53, 120.26, 114.62, 114.46, 55.94. Analysis: calc. for C_27_H_20_N_6_O_6_S (556.55): C 58.27, H 3.62, N 15.10%, found: C 58.43, H 3.74, N 14.89%.

##### 4.1.1.11 (*Z*)-2,6-dibromo-4-((2-(4-methoxyphenyl)-5-oxo-1-(4-(*N*-pyrimidin-2-ylsulfamoyl)phenyl)-1*H*-imidazole-4(5H)-ylidene)methyl)phenyl acetate (5k)

Yield: 69%, m.p. 239–241 °C. ^1^H-NMR (DMSo-*d*_6_, 400 MHz) *δ* (ppm): 11.98 (s, 1H, NH), 8.75 (s, 2H, Ar–H), 8.54 (d, *J* = 4.9 Hz, 2H, C3,5-H pyrimidine), 8.10–8.05 (m, 2H, Ar–H), 7.51 (d, *J* = 8.6 Hz, 2H, Ar–H), 7.40 (d, *J* = 8.9 Hz, 2H, Ar–H), 7.23 (s, 1H, CH=N), 7.10 (t, *J* = 4.9 Hz, 1H, C4-H pyrimidine), 6.94 (d, *J* = 8.9 Hz, 2H, Ar–H), 3.80 (s, 3H, OCH_3_), 2.44 (s, 3H, CH_3_). ^13^C-NMR (DMSo, 101 MHz) *δ*: 172.47, 169.51, 167.68, 162.74, 161.84, 157.17, 146.58, 140.46, 140.01, 138.55, 135.80, 135.71, 131.41, 129.11, 128.68, 122.60, 120.29, 117.91, 114.63, 56.02, 21.52. Analysis: calc. for C_29_H_21_Br_2_N_5_O_6_S (727.38): C 47.89, H 2.91, N 9.63%, found: C 48.03, H 3.04, N 9.51%.

##### 4.1.1.12 (*Z*)-4-(2-(4-methoxyphenyl)-5-oxo-4-(3,4,5-trimethoxybenzylidene)-4,5-dihydro-1*H*-imidazole-1-yl)-*N*-(pyrimidin-2-yl)benzenesulfonamide (5l)

Yield: 66%, m.p. 247–249 °C. ^1^H-NMR (DMSo-*d*_6_, 400 MHz) *δ* (ppm): 8.48 (d, *J* = 4.9 Hz, 2H, C3,5-H pyrimidine), 8.06–8.01 (m, 2H, Ar–H), 7.83 (s, 2H, Ar–H), 7.46 (d, *J* = 8.6 Hz, 2H, Ar–H), 7.42 (d, *J* = 8.9 Hz, 2H, Ar–H), 7.18 (s, 1H, CH=N), 7.00 (t, *J* = 4.8 Hz, 1H, C4-H pyrimidine), 6.91 (d, *J* = 9.0 Hz, 2H, Ar–H), 3.86 (s, 6H, 2OCH_3_), 3.79 (s, 3H, OCH_3_), 3.76 (s, 3H, OCH_3_). ^13^C-NMR (DMSo, 101 MHz) *δ* 169.82, 164.06, 162.34, 159.46, 158.61, 153.26, 140.25, 137.87, 131.11, 130.48, 130.10, 129.41, 128.97, 128.38, 127.50, 120.76, 117.62, 115.48, 114.54, 110.36, 110.15, 60.69, 56.35, 55.95. Analysis: calc. for C_30_H_27_N_5_O_7_S (601.63): C 59.89, H 4.52, N 11.64%, found: C 60.08, H 4.40, N 11.52%.

##### 4.1.1.13 (*Z*)-4-(4-(furan-2-ylmethylene)-5-oxo-2-phenyl-4,5-dihydro-1*H*-imidazole-1-yl)-*N*-(pyrimidin-2-yl)benzenesulfonamide (6a)

Yield: 54%, m.p. 291–293 °C. ^1^H-NMR (DMSo-*d*_6_, 400 MHz) *δ* (ppm): 11.98 (s, 1H, NH), 8.52 (d, *J* = 4.9 Hz, 2H, C3,5-H pyrimidine), 8.07–8.04 (m, 1H, C5-H furan), 8.03 (d, *J* = 8.7 Hz, 2H, Ar–H), 7.58 (d, *J* = 3.5 Hz, 1H, C3-H furan), 7.52–7.48 (m, 1H, Ar–H), 7.47 (d, *J* = 2.0 Hz, 1H, Ar–H), 7.45 (t, *J* = 2.6 Hz, 2H, Ar–H), 7.43 (d, *J* = 1.4 Hz, 1H, Ar–H), 7.35 (t, *J* = 7.7 Hz, 2H, Ar–H), 7.15 (s, 1H, Ar–H), 7.09 (t, *J* = 4.9 Hz, 1H, C4-H pyrimidine), 6.85–6.79 (m, 1H, C4-H furan). Analysis: calc. for C_24_H_17_N_5_O_4_S (471.49): C 61.14, H 3.63, N 14.85%, found: C 60.93, H 3.76, N 15.02%.

##### 4.1.1.14 (*Z*)-4-(4-(furan-2-ylmethylene)-2-(4-methoxyphenyl)-5-oxo-4,5-dihydro-1*H*-imidazole-1-yl)-*N*-(pyrimidin-2-yl)benzenesulfonamide (6b)

Yield: 61%, m.p. 278–280 °C. ^1^H-NMR (DMSo-*d*_6_, 400 MHz) *δ* (ppm): 12.04 (s, 1H, NH), 8.53 (d, *J* = 4.9 Hz, 2H, C3,5-H pyrimidine), 8.06 (d, *J* = 8.5 Hz, 2H, Ar–H), 8.02 (d, *J* = 1.5 Hz, 1H, C5-H furan), 7.57 (d, *J* = 3.4 Hz, 1H, C3-H furan), 7.48 (d, *J* = 8.5 Hz, 2H, Ar–H), 7.40 (d, *J* = 8.8 Hz, 2H, Ar–H), 7.09 (t, *J* = 4.9 Hz, 1H, C4-H pyrimidine), 7.07 (s, 1H, CH=N), 6.90 (d, *J* = 8.9 Hz, 2H, Ar–H), 6.81 (dd, *J* = 3.4, 1.6 Hz, 1H, C4-H furan), 3.78 (s, 3H, OCH_3_). ^13^C-NMR (DMSo, 101 MHz) *δ*: 172.48, 169.22, 162.31, 159.28, 158.78, 157.26, 151.02, 147.49, 140.58, 138.79, 135.98, 131.25, 129.07, 128.57, 120.69, 119.63, 114.44, 114.41, 114.36, 55.95. Analysis: calc. for C_25_H_19_N_5_O_5_S (501.51): C 59.87, H 3.82, N 13.96%, found: C 60.03, H 4.96, N 13.84%.

## Conflicts of interest

The authors report no conflicts of interest.

## Supplementary Material

RA-014-D4RA03157A-s001
