# Supplementary material for: Synthesis and biological research of new imidazolone-sulphonamide-pyrimidine hybrids as potential EGFR-TK inhibitors and apoptosis-inducing agents
Source: RSC Adv. 2024 Jun 24;14(28):20120–9. doi: 10.1039/d4ra03157a (PMC11194663; doi:10.1039/d4ra03157a)
Supplement: RA-014-D4RA03157A-s001 [file RA-014-D4RA03157A-s001.pdf]

Electronic Supplementary Material (ESI) for RSC Advances.
This journal is © The Royal Society of Chemistry 2024

Synthesis and Biological Research of New Imidazolone-Sulphonamide-Pyrimidine Hybrids as Potential EGFR-TK Inhibitors and Apoptosis-Inducing Agents

Dalal Nasser Binjawhar [1], Hanadi A. Katouah [2], Najla A. Alshaye [1], Jawaher Alharthi [3], Ghadi Alsharif [4,5], Fahmy Gad Elsaid [6], Eman Fayad [3], and Ali. H. Abu Almaaty [7, *]

[1] *Department of Chemistry, College of science, Princee Nourah bint Abdulrahman University, P.O. Box 84428, Riyadh 11671, Saudi Arabia*

[2] *Chemistry Department, College of Science, Umm Al-Qura University, 21955, Makkah, Saudi Arabia*

[3] *Department of Biotechnology, College of Sciences, Taif University, P.O. Box 11099, Taif 21944, Saudi Arabia*

[4] *Department of Clinical Laboratory Sciences, College of Applied Medical Sciences, King Saud Bin Abdulaziz University for Health Sciences, P.O.Box 9515 Jeddah 21423,Saudi Arabia*

[5] *Department of Biomedical Research, King Abdullah International Medical Research Center, 21423 Jeddah, Saudi Arabia*

[6] *Department of Biology, College of Science, King Khalid University, PO Box 960, Asir, Abha, 61421, Saudi Arabia*

[7] *Zoology Department, Faculty of Science, Port Said University, Port Said 42526, Egypt*

---

* *To whome correspondence should be addressed*

Ali. H. Abu Almaaty, PhD. Zoology Department, Faculty of Science, Port Said University, Port Said 42526, Egypt

E-mail address: aliabuelmaaty8@gmail.com

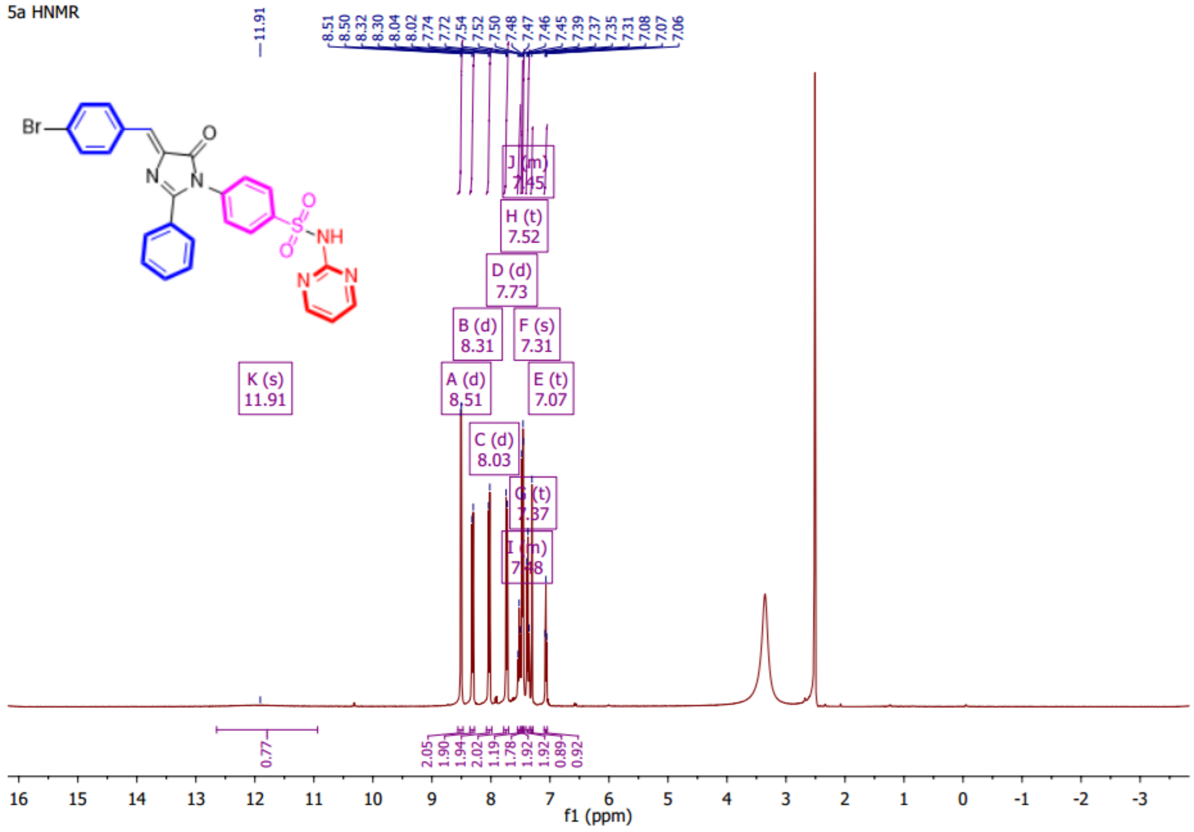

Figure S1: ^{1}H-NMR spectrum of compound **5a**

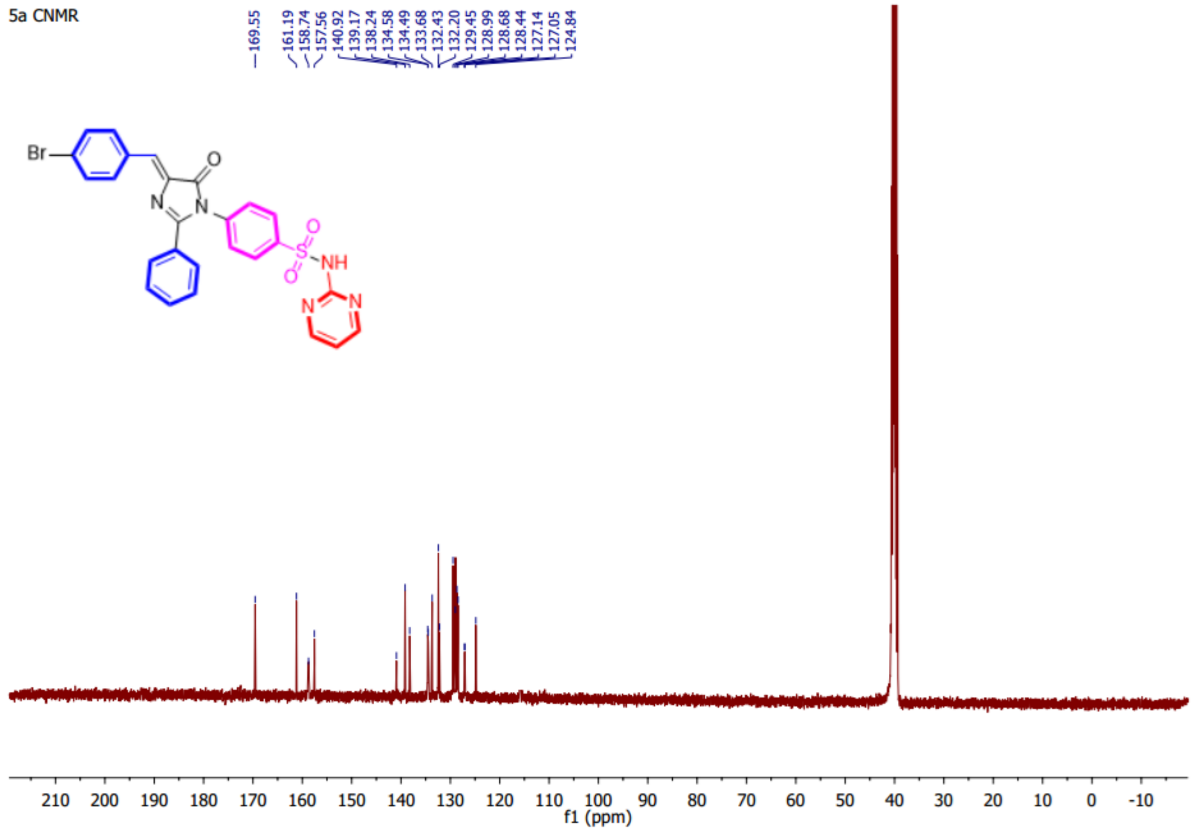

Figure S2: ^{13}C-NMR spectrum of compound **5a**

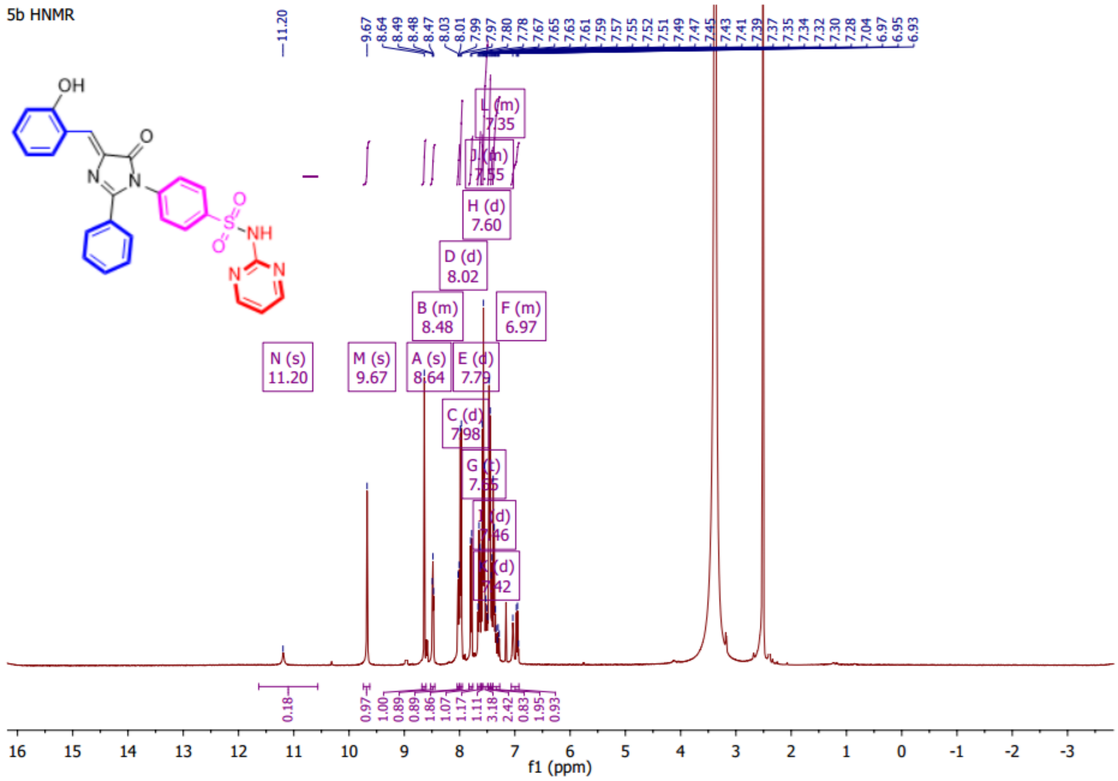

Figure S3: ^{1}H-NMR spectrum of compound **5b**

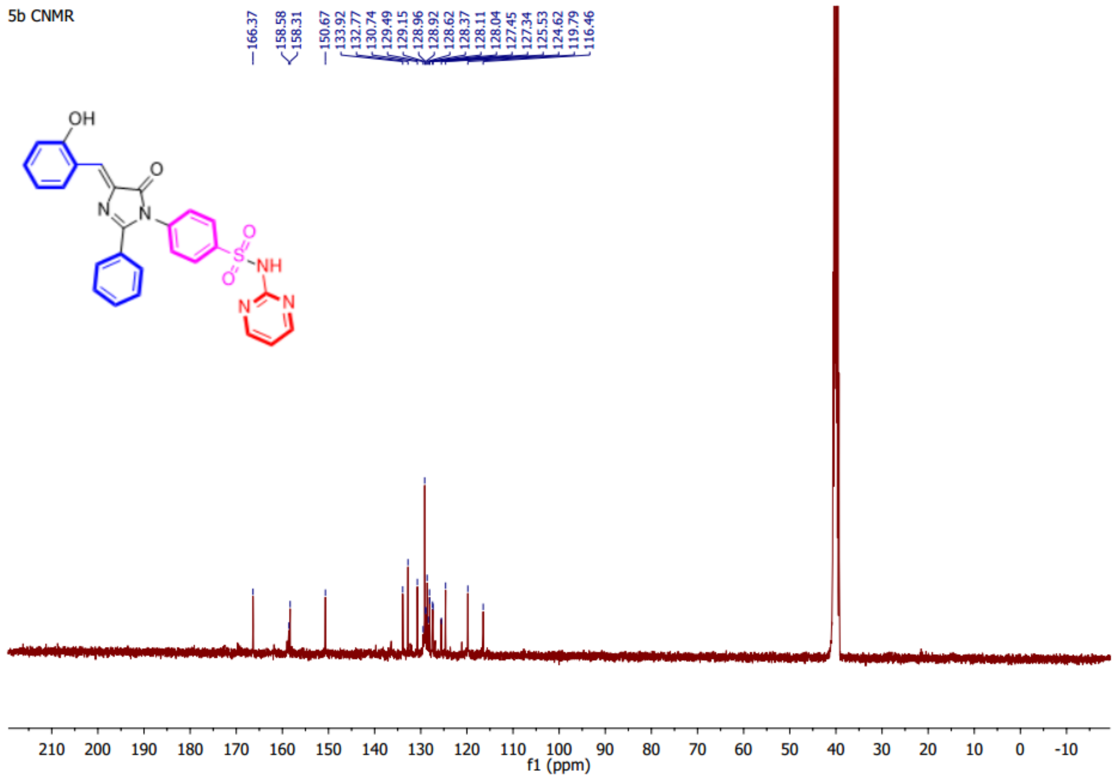

Figure S4: ^{13}C-NMR spectrum of compound **5b**

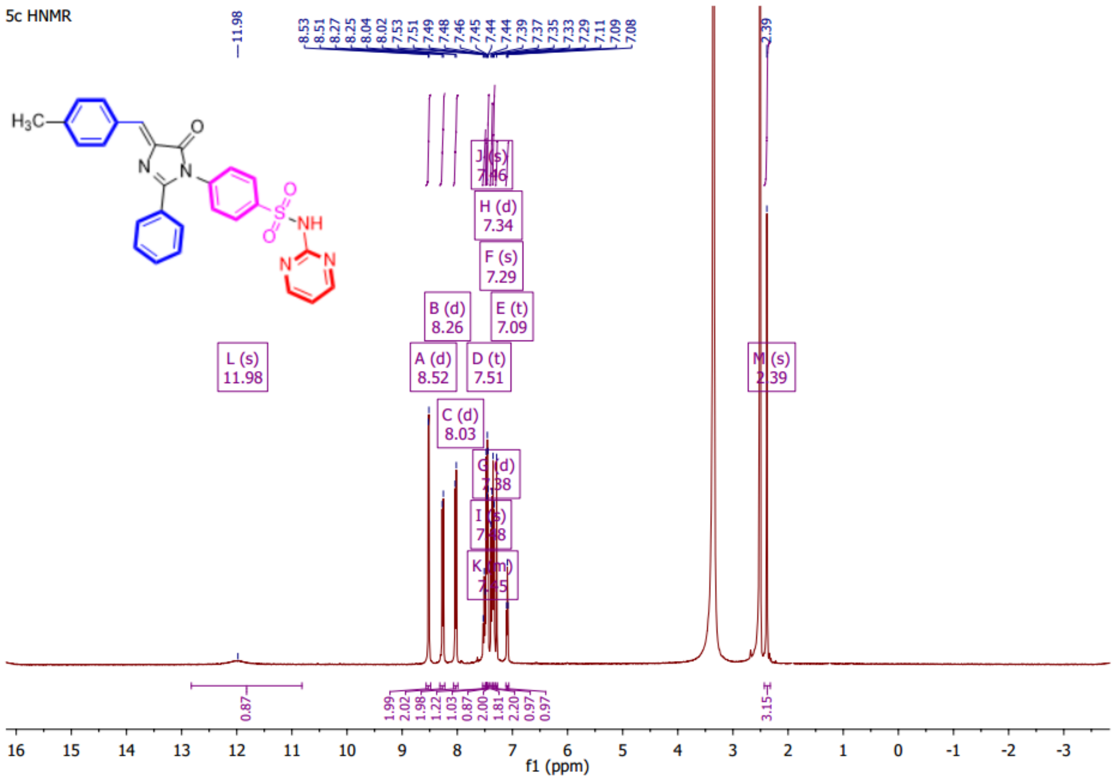

Figure S5: ^{1}H-NMR spectrum of compound **5c**

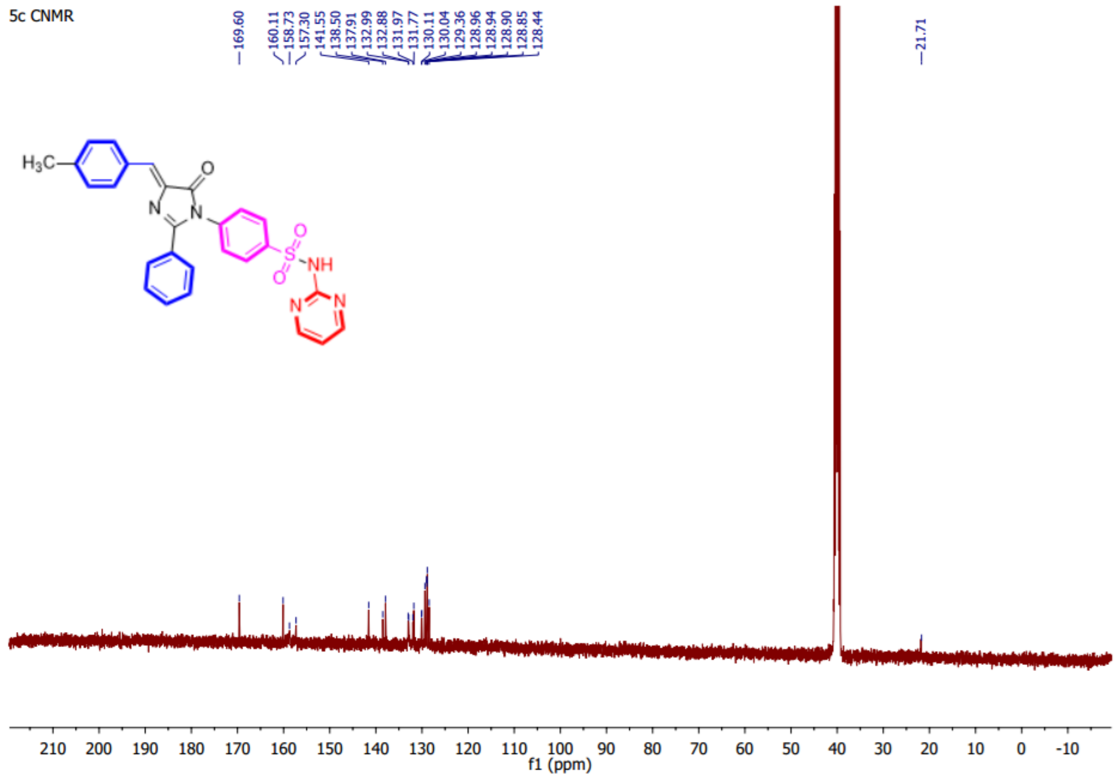

Figure S6: ^{13}C-NMR spectrum of compound **5c**

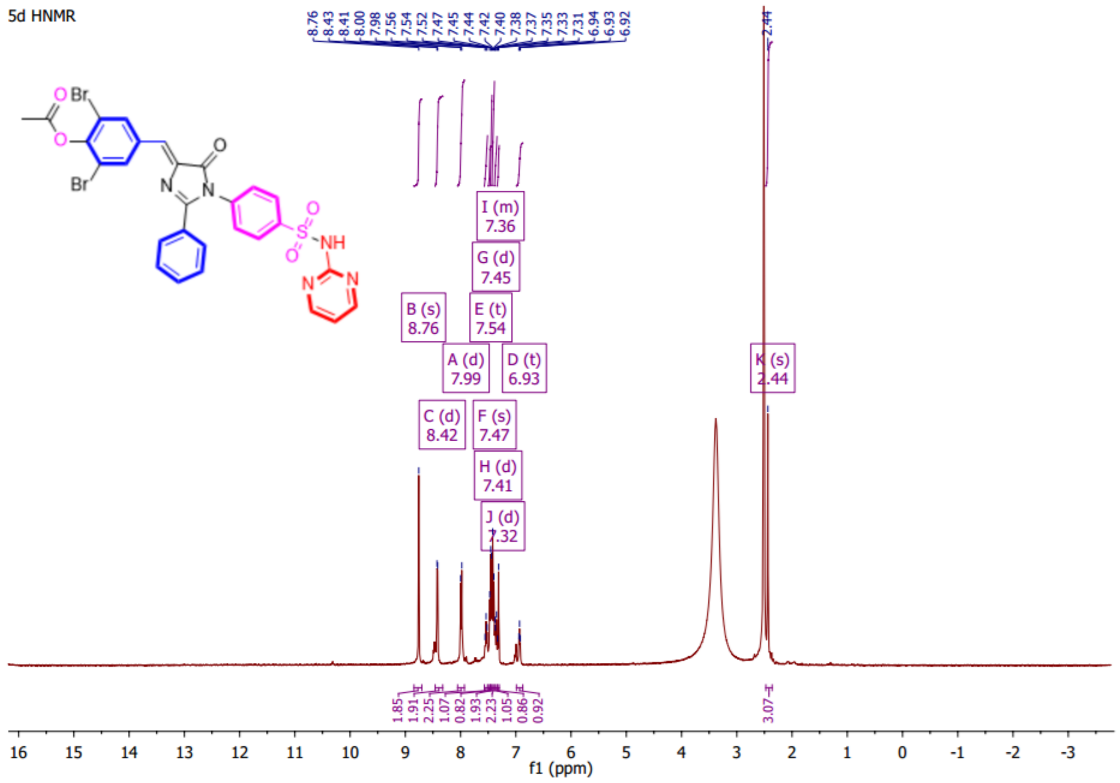

Figure S7: ^{1}H-NMR spectrum of compound **5d**

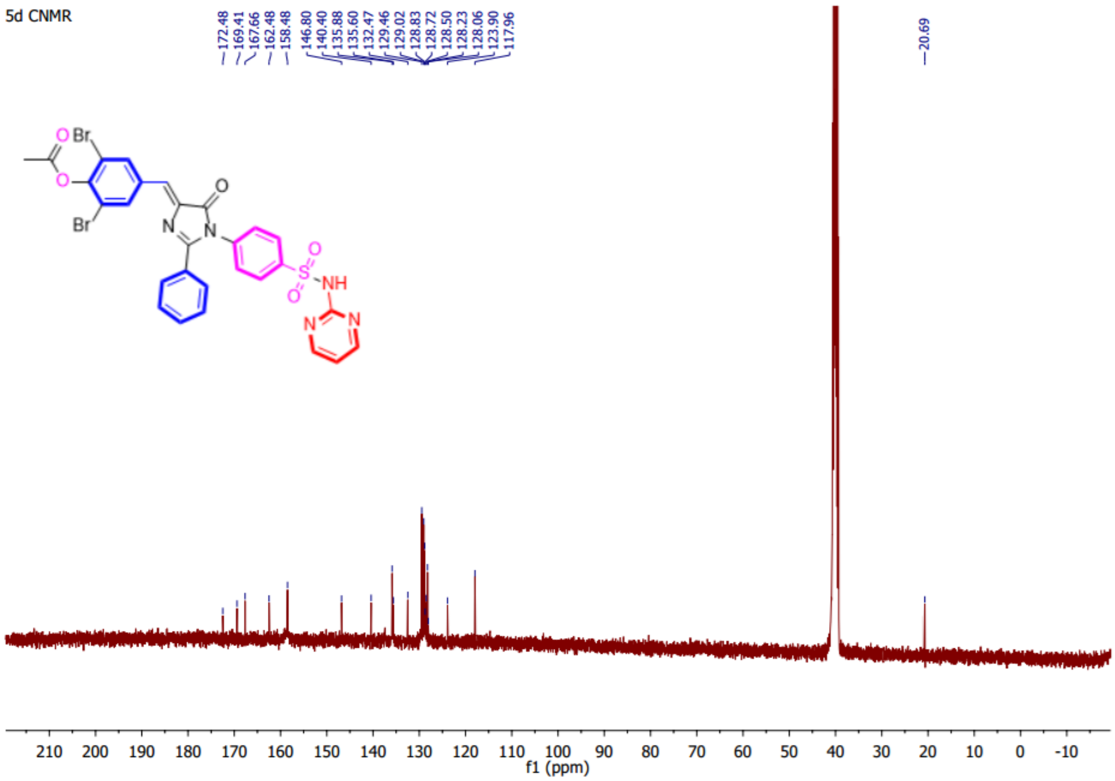

Figure S8: ^{13}C-NMR spectrum of compound **5d**

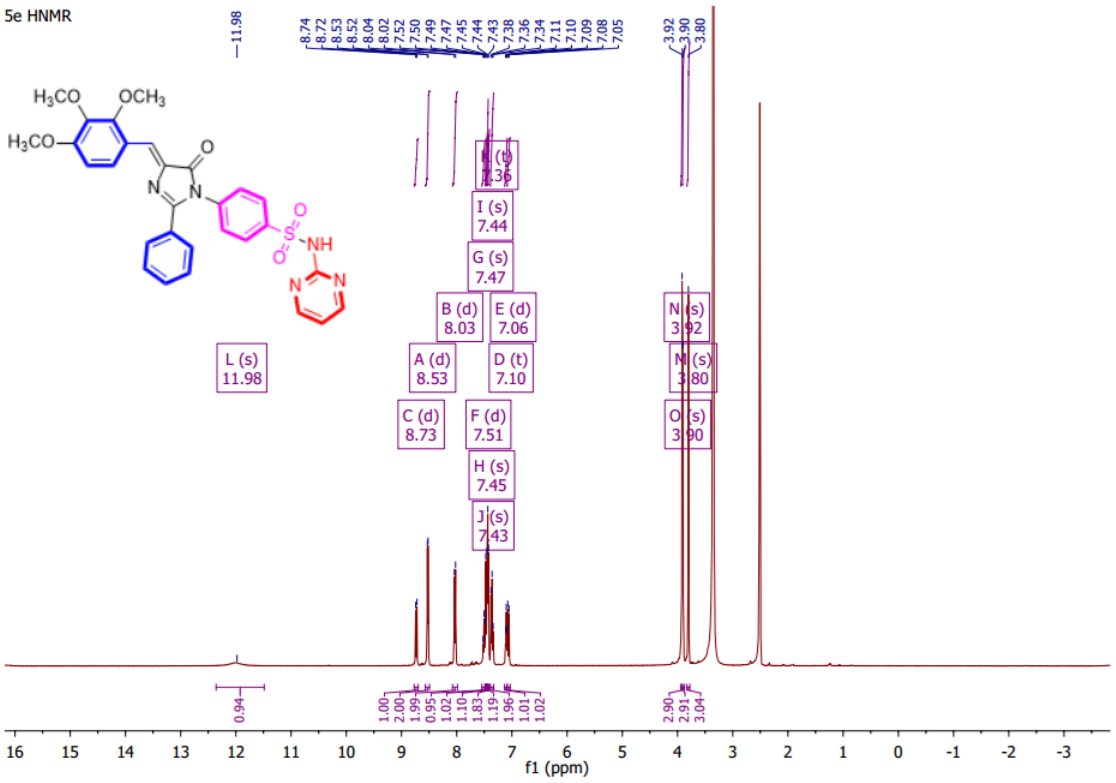

Figure S9: ^{1}H-NMR spectrum of compound **5e**

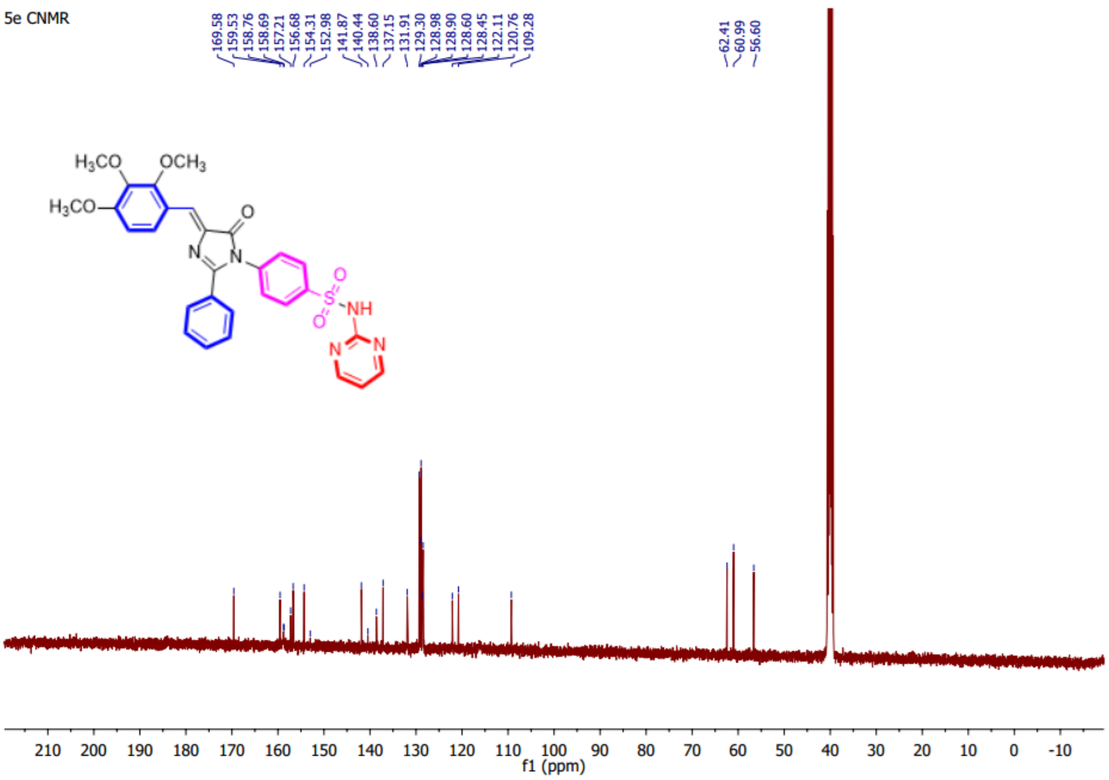

Figure S10: ^{13}C-NMR spectrum of compound **5e**

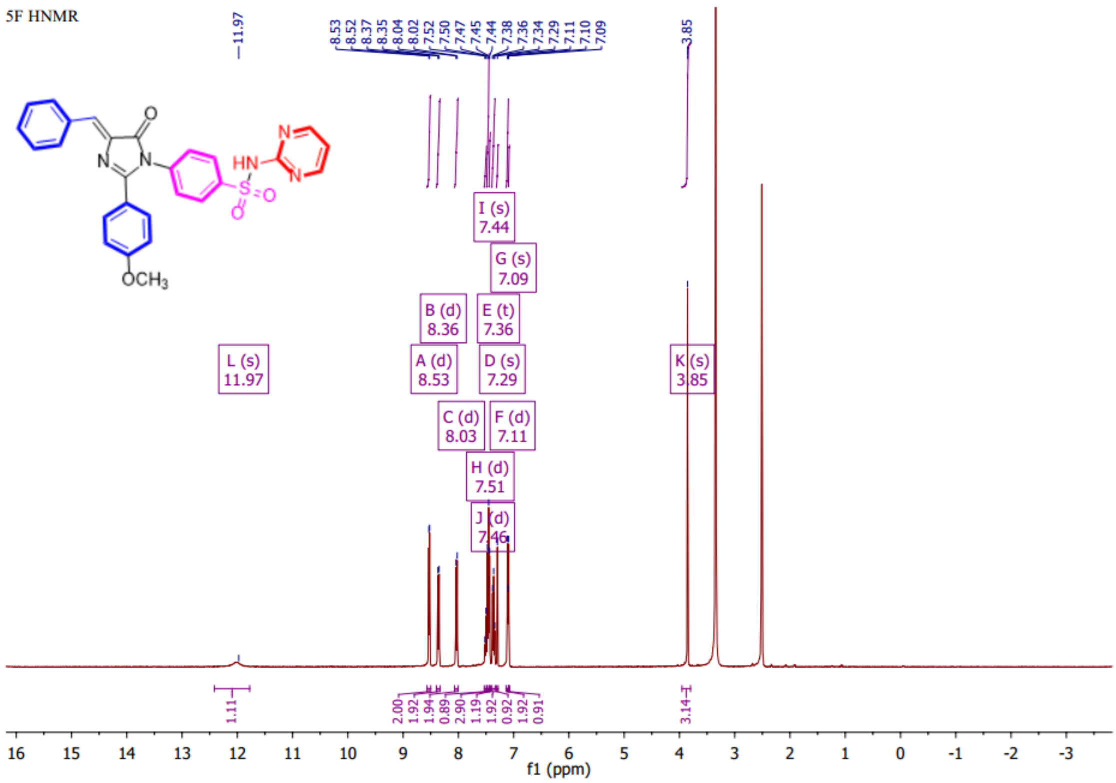

Figure S11: ^{1}H-NMR spectrum of compound **5f**

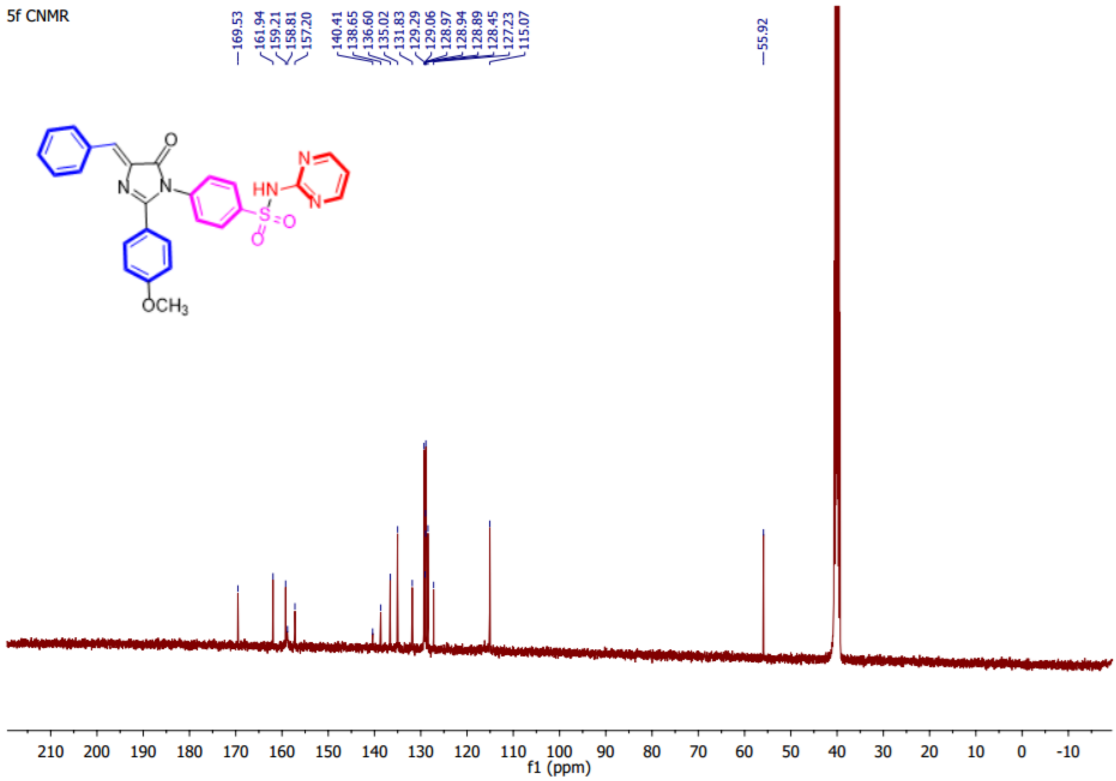

Figure S12: ^{13}C-NMR spectrum of compound **5f**

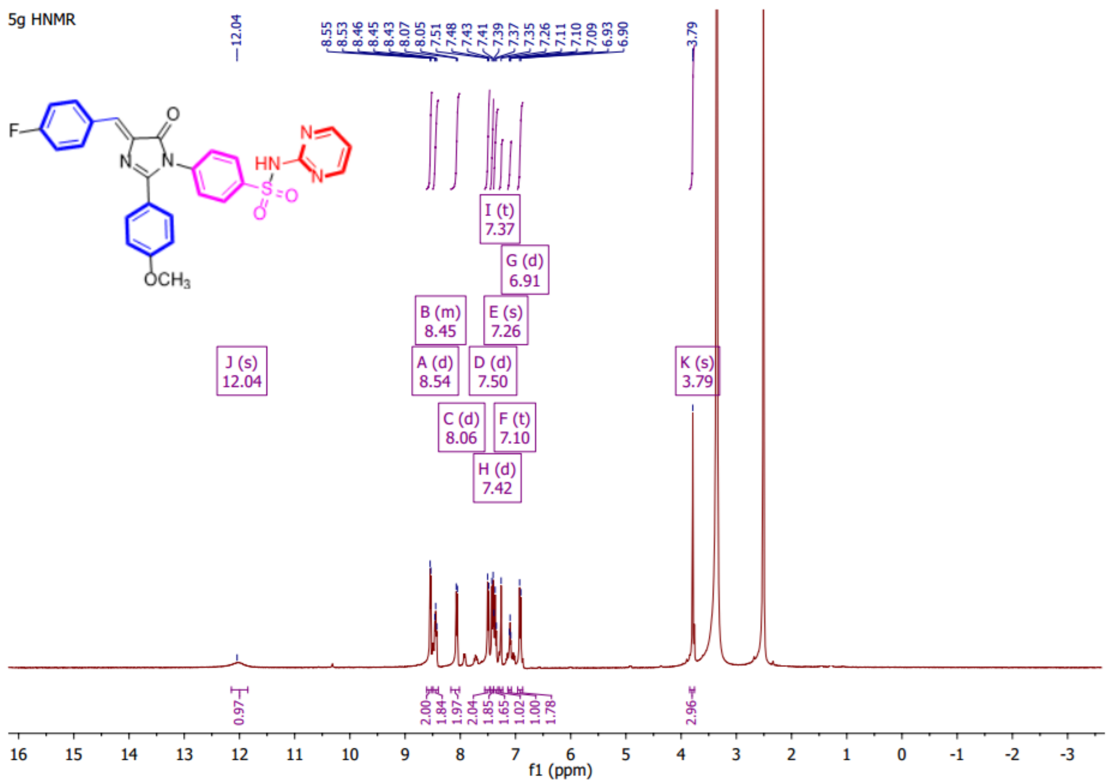

Figure S13: ^{1}H-NMR spectrum of compound **5g**

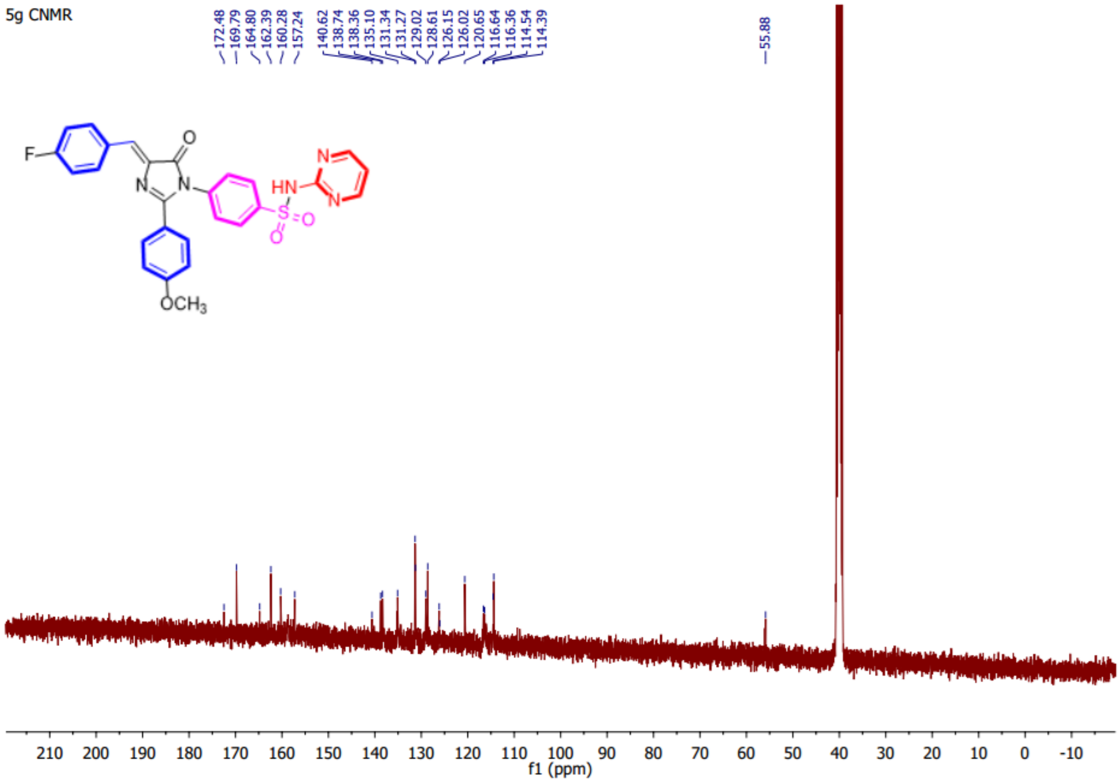

Figure S14: ^{13}C-NMR spectrum of compound **5g**

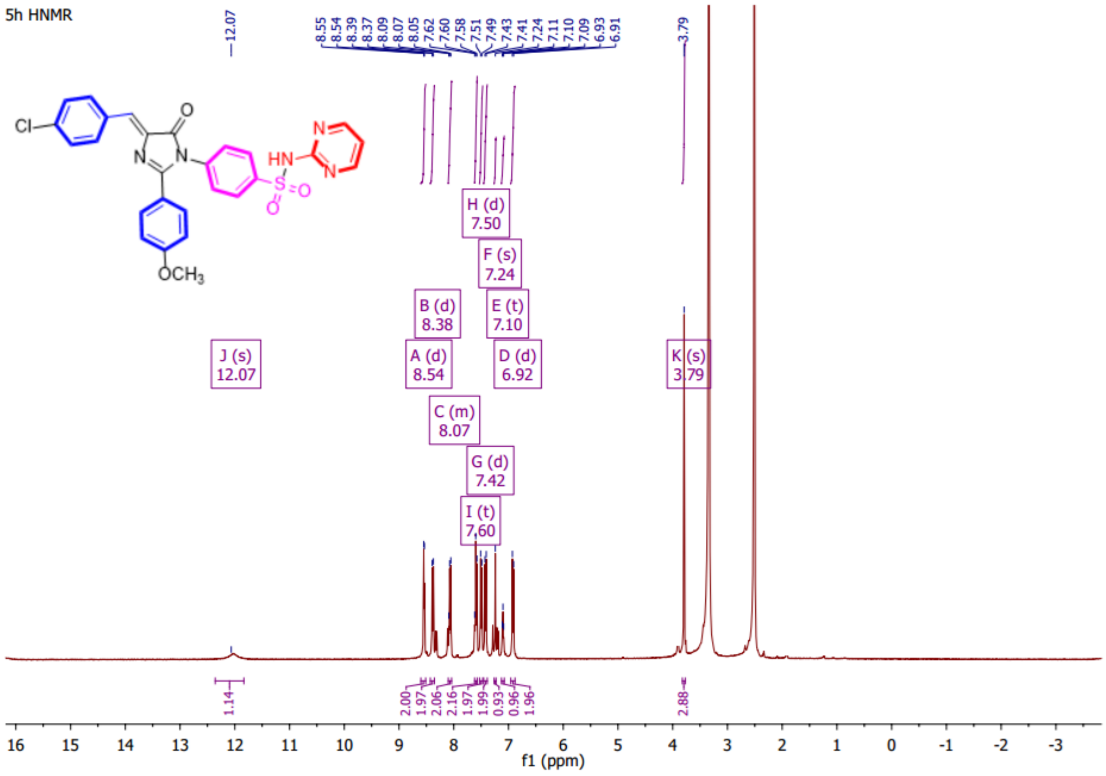

Figure S15: ^{1}H-NMR spectrum of compound **5h**

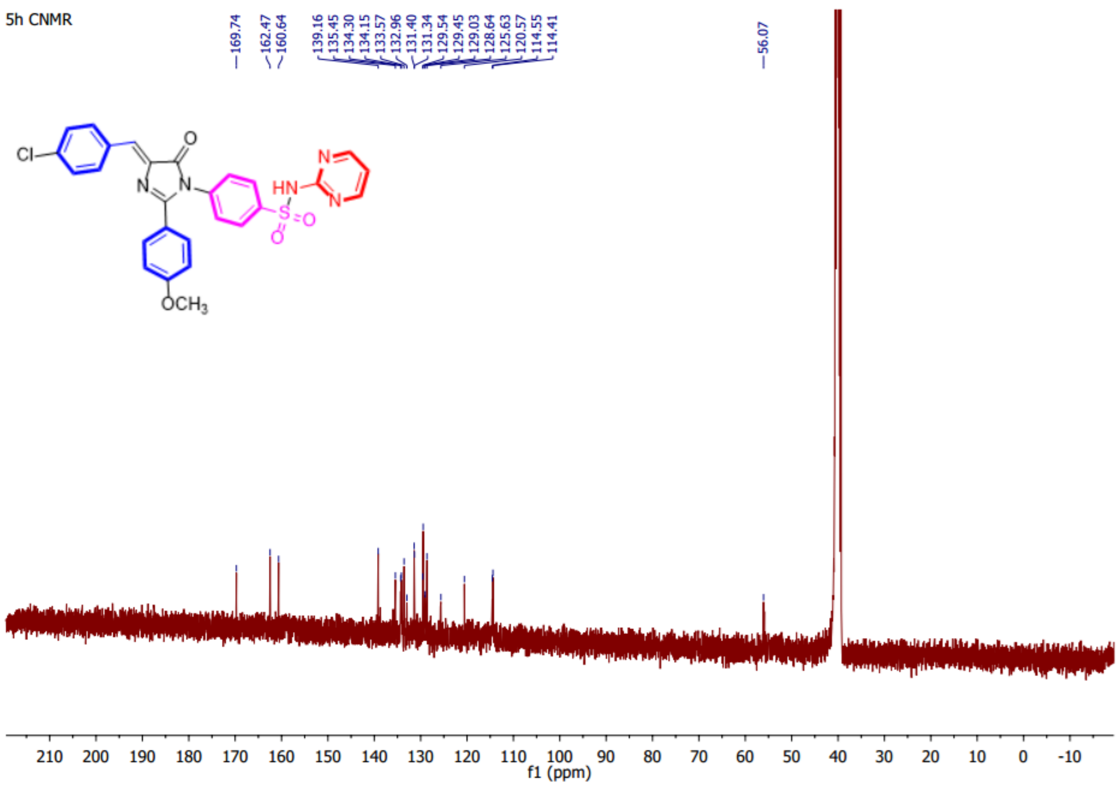

Figure S16: ^{13}C-NMR spectrum of compound **5h**

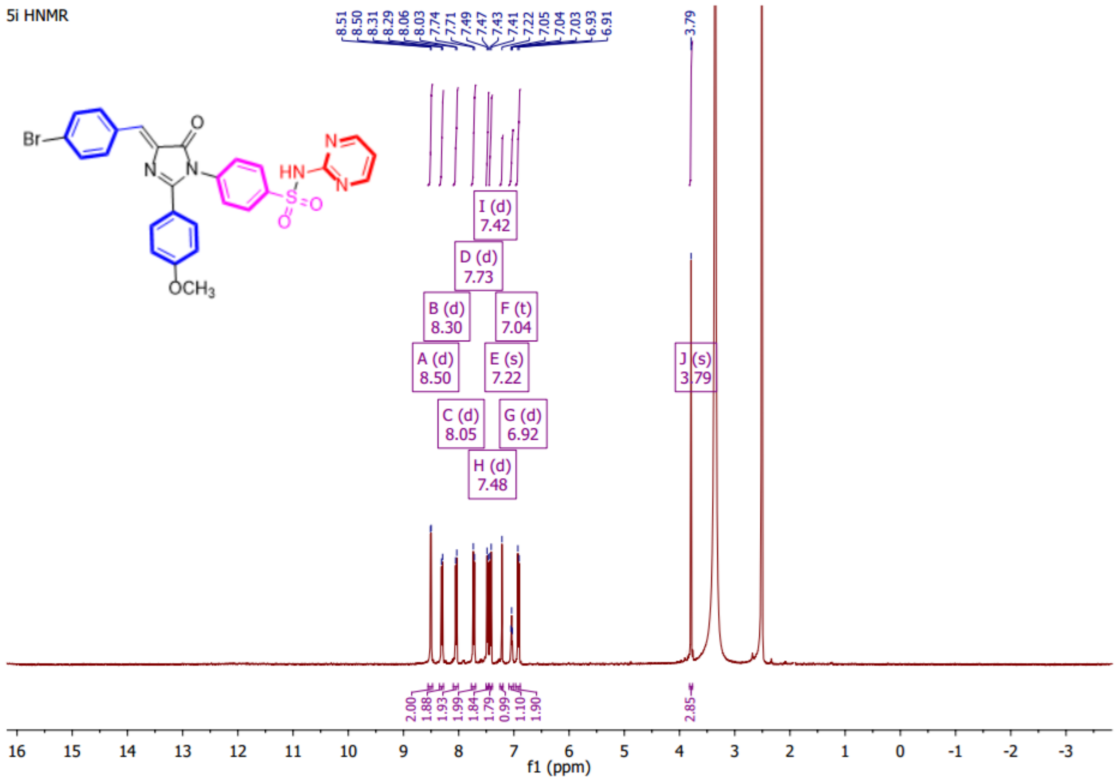

Figure S17: ^{1}H-NMR spectrum of compound **5i**

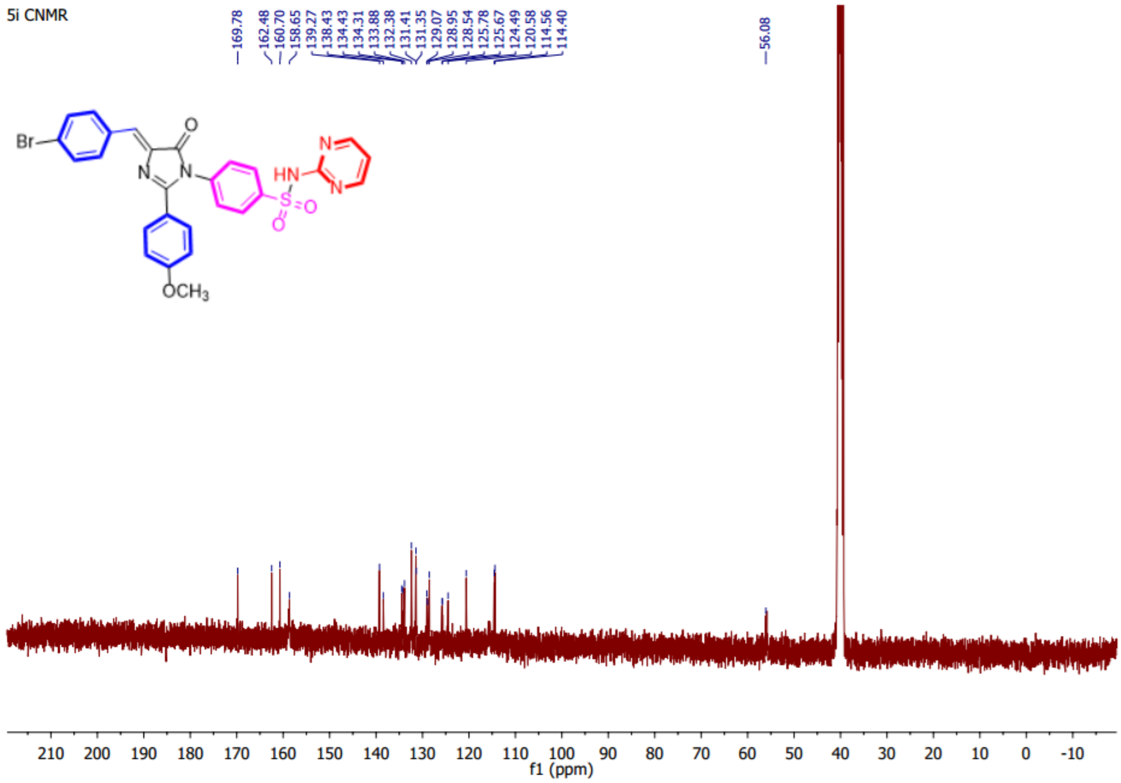

Figure S18: ^{13}C-NMR spectrum of compound **5i**

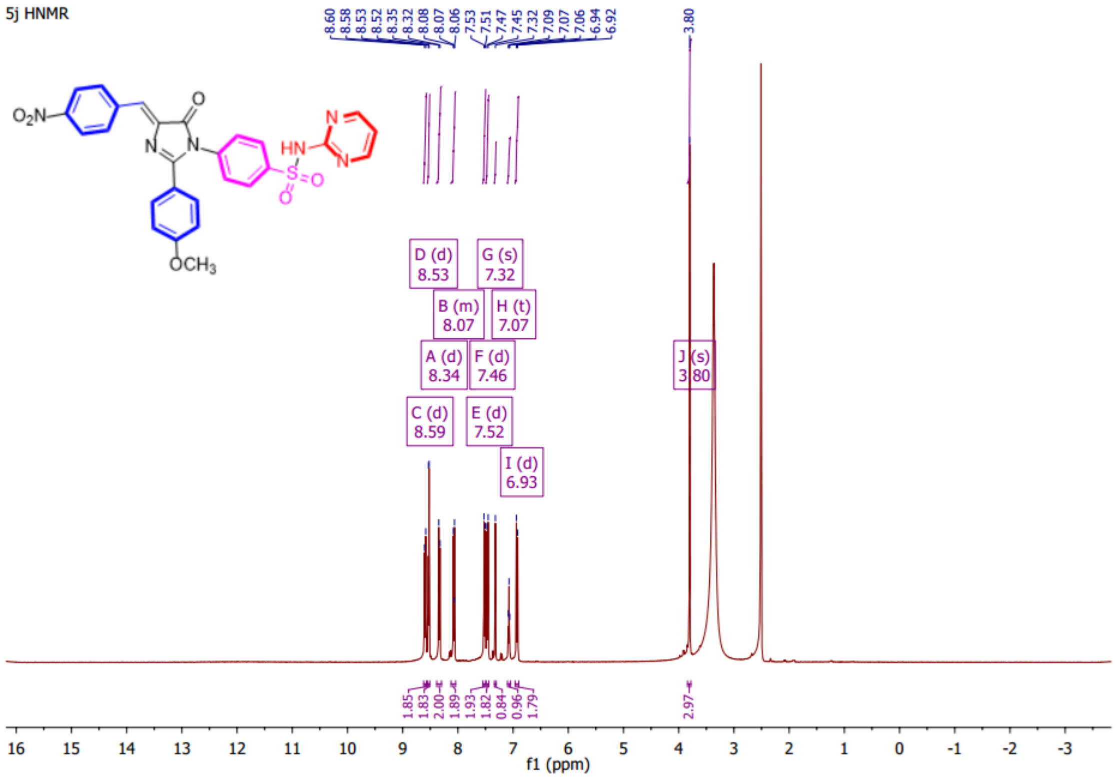

Figure S19: ^{1}H-NMR spectrum of compound **5j**

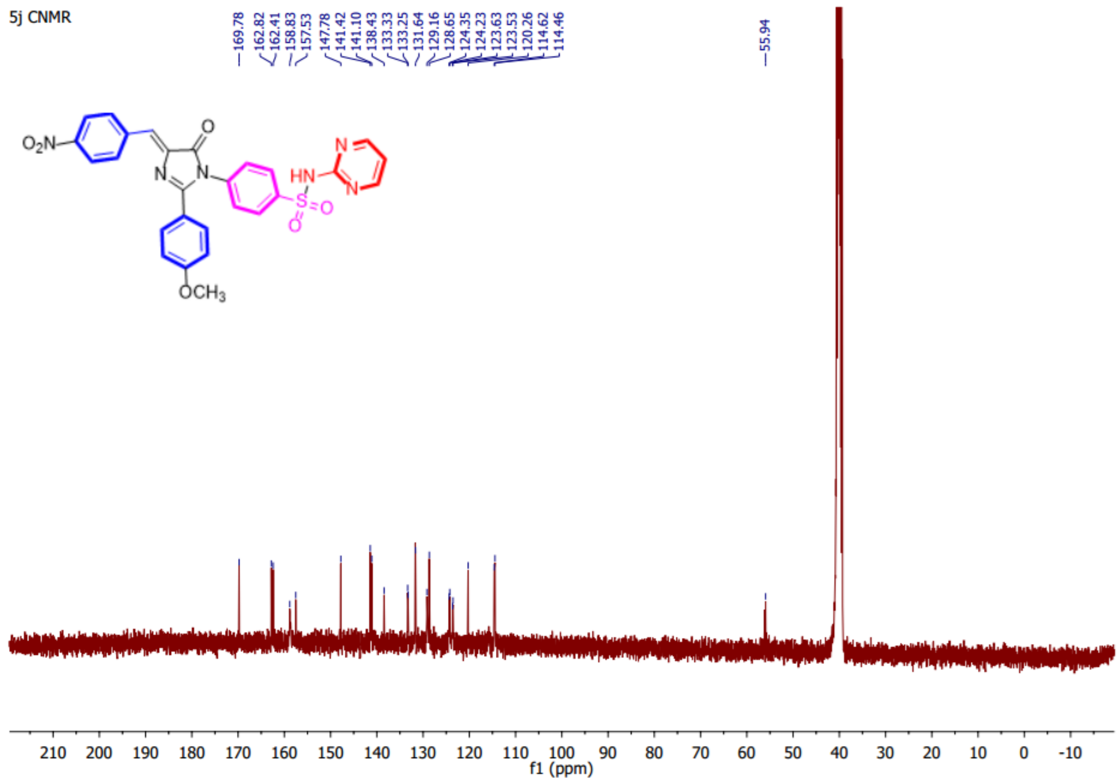

Figure S20: ^{13}C-NMR spectrum of compound **5j**

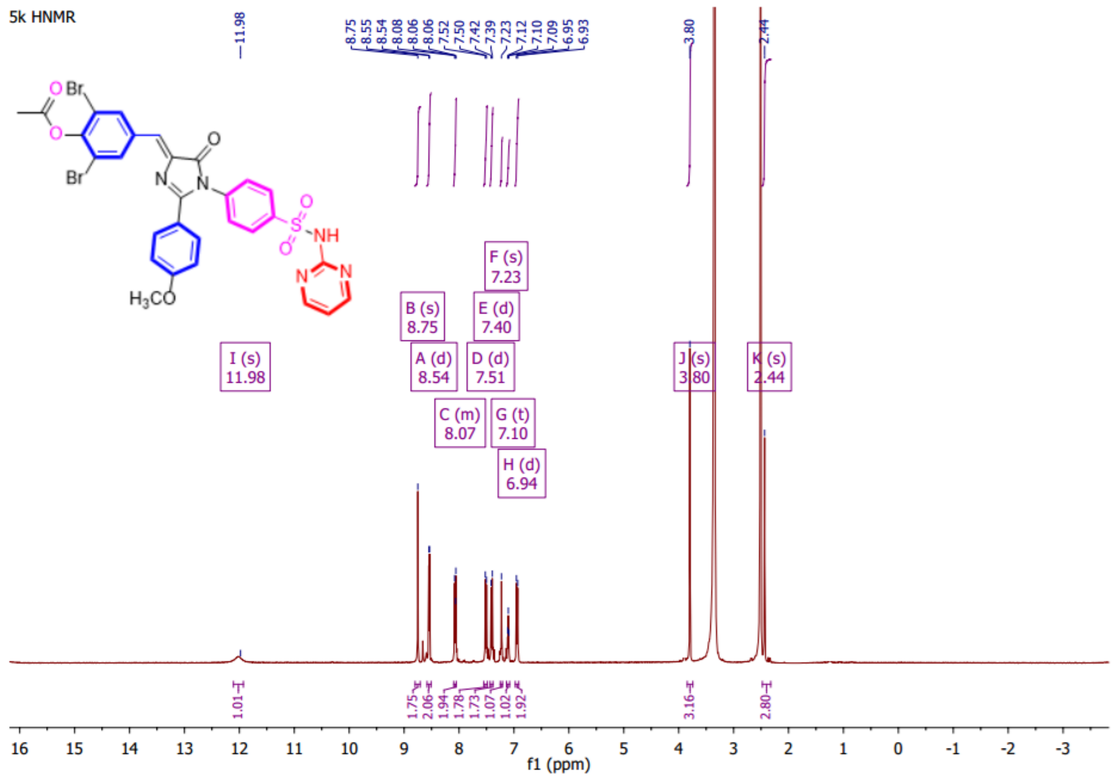

Figure S21: ^{1}H-NMR spectrum of compound **5k**

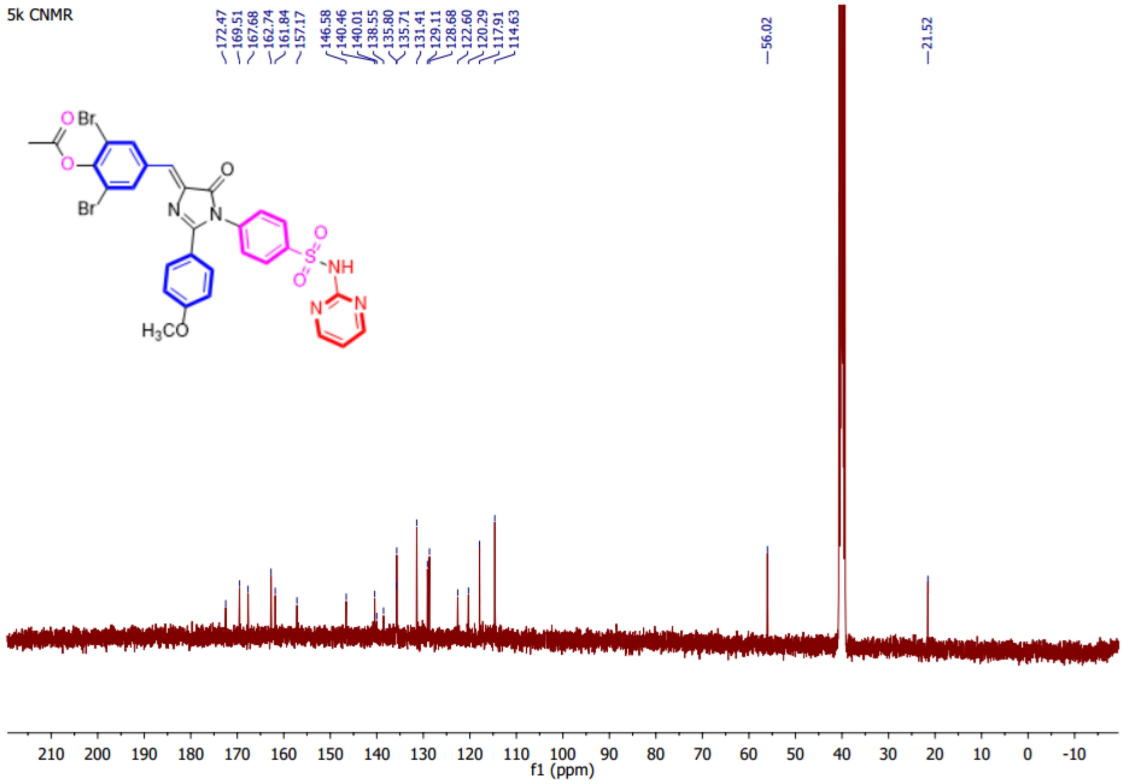

Figure S22: ^{13}C-NMR spectrum of compound **5k**

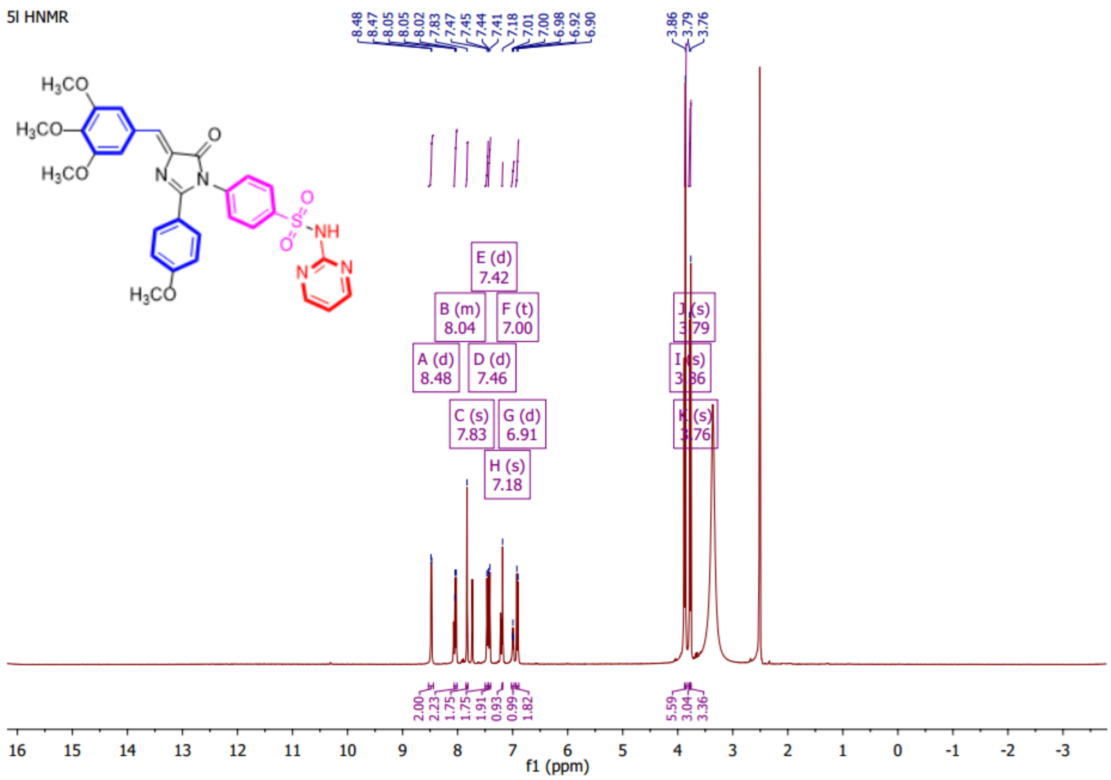

Figure S23: ^{1}H-NMR spectrum of compound **5l**

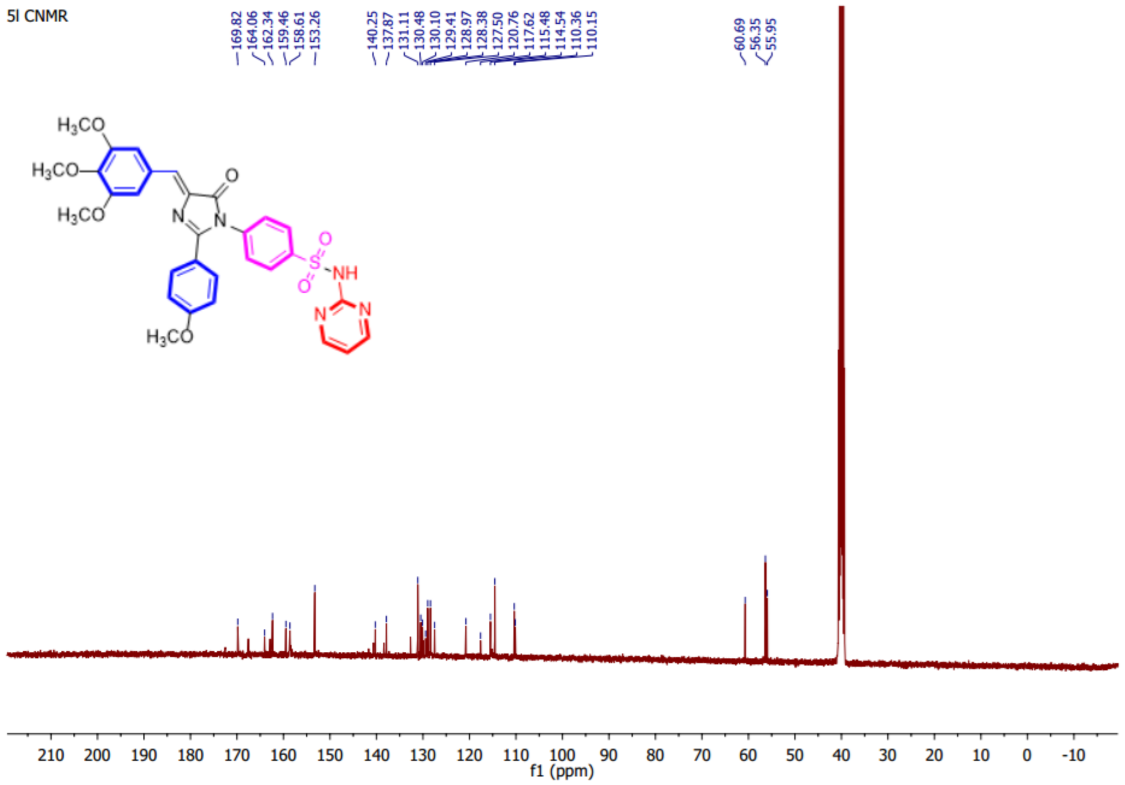

Figure S24: ^{13}C-NMR spectrum of compound **5l**

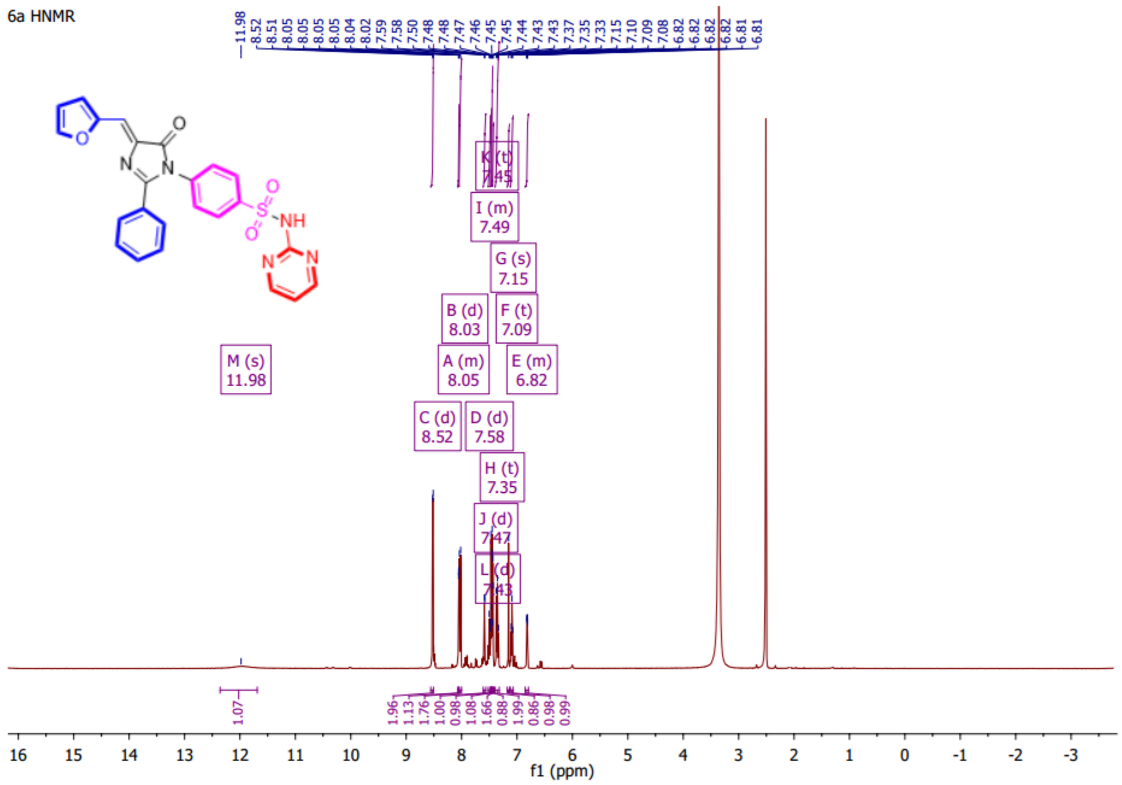

Figure S25: ^{1}H-NMR spectrum of compound **6a**

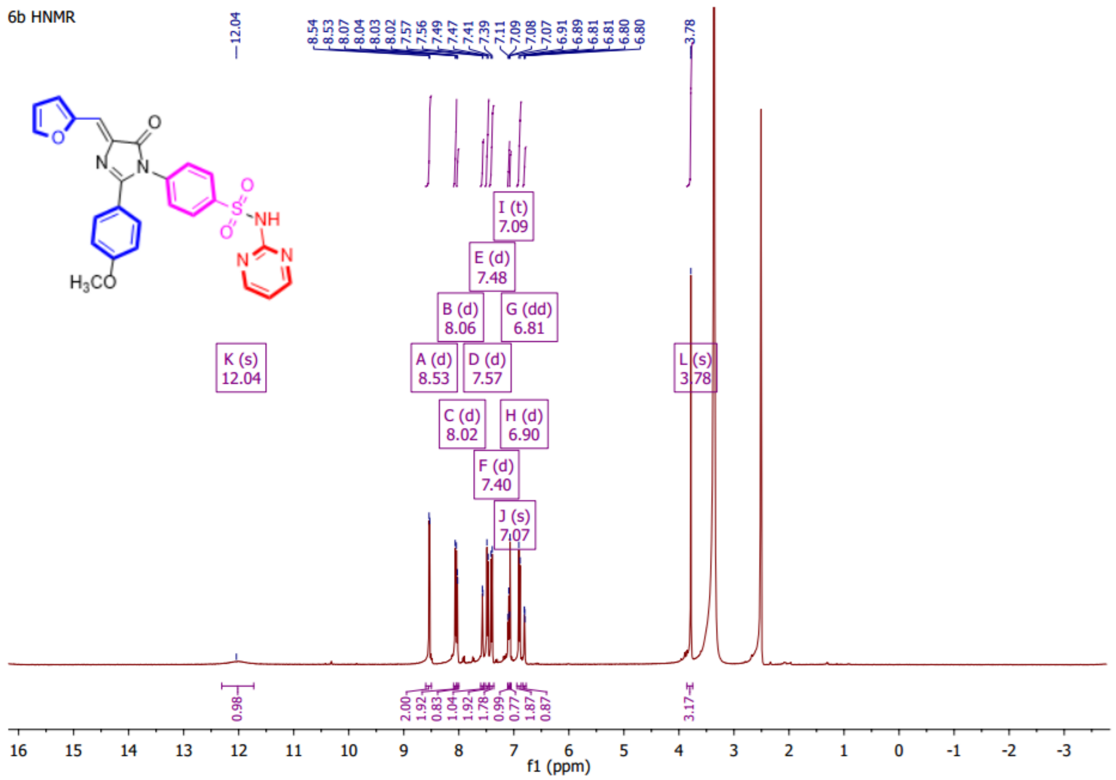

Figure S26: ^{1}H-NMR spectrum of compound **6b**

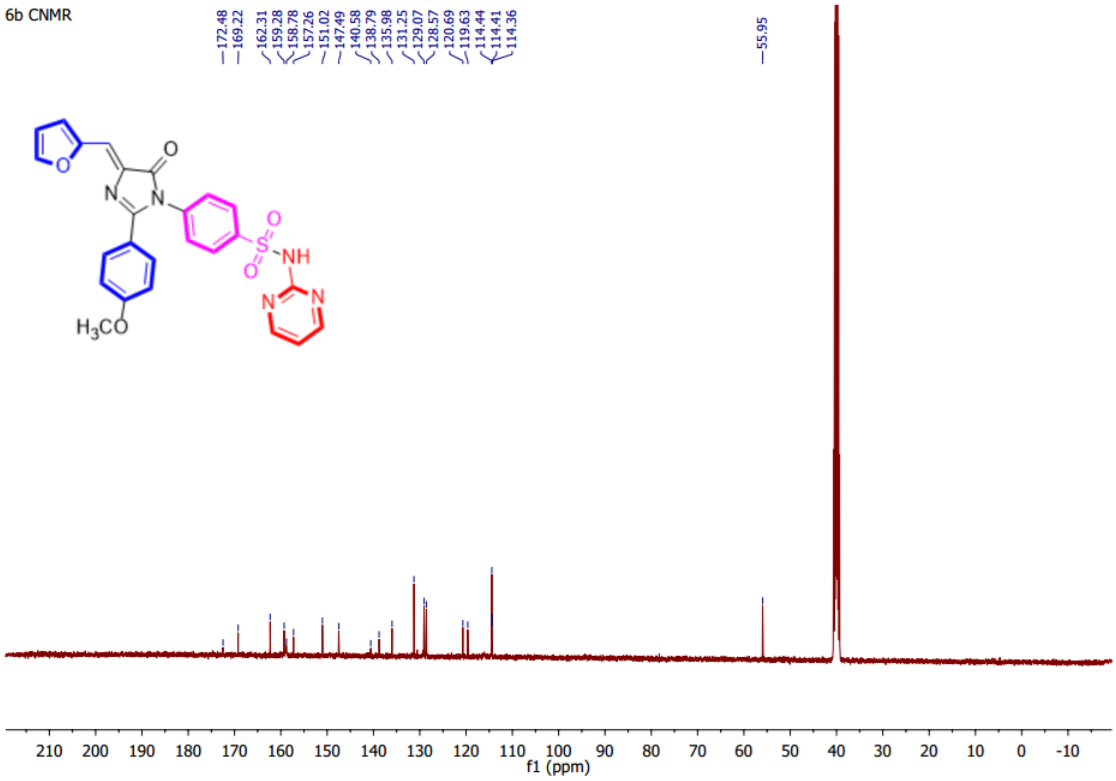

Figure S27: ^{13}C-NMR spectrum of compound **6b**

Appendix A

S4.2. Biological Studies

S4.2.1. Cytotoxic activity evaluation

To measure the cytotoxic actvity of the synthesized derivatives **5a-l** and **6a,b** in breast adenocarcinoma (MCF-7) cell line. Cell viability assay was assessed using MTT assay method. Cells at density of 1 x 10^4 were seeded in a 96-well plate at 37 ^oC for 24 h under 5% CO_2. After incubation, the cells were treated with different concentrations of the test imidazolone hybrids **5a-l** and **6a,b** and incubated for 24 h, then 20 µl of MTT solution at 5 mg/mL was applied and incubated for 4 h at 37 °C. Dimethyl sulphoxide (DMSO) in volume of 100 µl was added to each well to dissolve the purple formazan that had formed. The color intensity of the formazan product, which represents the growth condition of the cells, is quantified by using an ELISA plate reader (EXL 800, USA) at 570 nm absorbance. The experimental conditions were carried out with at least three replicates, and the experiments were repeated at least three times.

S4.2.2. EGFR kinase Assay

Compounds **5h**, **5j**, **6b** and Lapatinib were evaluated for their EGFR kinase inhibitory activity according to manufacturer's instructions using # BPS Bioscience *EGFR Kinase Assay Kit* Catalog # 40321.

BPS Bioscience

6042 Cornerstone Court W, Ste B
San Diego, CA 92121
Tel: 1.858.829.3082
Fax: 1.858.481.8694
Email: info@bpsbioscience.com

Data Sheet
EGFR Kinase Assay Kit
Catalog # 40321

DESCRIPTION: The epidermal growth factor receptor (EGFR; ErbB-1; HER1) is the cell-surface receptor for members of the epidermal growth factor family. Overexpression and/or hyperactivation of EGFR kinase is associated with several human cancers such as lung, glioblastoma, and epithelian tumors of the neck and head, leading to the development of anticancer therapeutics targeting EGFR. The *EGFR Kinase Assay Kit* is designed to measure EGFR Kinase activity for screening and profiling applications using Kinase-Glo® MAX as a detection reagent. The EGFR Kinase Assay Kit comes in a convenient 96-well format, with enough purified recombinant EGFR enzyme, EGFR substrate, ATP and kinase assay buffer for 100 enzyme reactions.

COMPONENTS:

Catalog #	Reagent	Amount	Storage	
40187	EGFR (wild type)	2 µg	-80°C	***Avoid multiple freeze/ thaw cycles!***
	5x Kinase assay buffer	1.5 ml	-20°C	
	ATP (500 µM)	100 µl	-20°C	
40217	50x PTK substrate Poly(Glu:Tyr 4:1)	100 µl	-20°C	
	96-well plate, white	1	Room Temp.	

MATERIALS OR INSTRUMENTS REQUIRED BUT NOT SUPPLIED:
Kinase-Glo MAX (Promega #V6071)
Dithiothreitol (DTT, 1 M; optional)
Microplate reader capable of reading luminescence
Adjustable micropipettor and sterile tips
30°C incubator

APPLICATIONS: Useful for studying enzyme kinetics and screening small molecular inhibitors for drug discovery and HTS applications.

STABILITY: Up to 6 months when stored as recommended.

REFERENCE:

Nakamura, J.L. *Expert Opin. Ther. Targets* **11(4)**:463-472 (2007)

S4.2.3. Cell cycle analysis of compound 6b

Cell cycle analysis in MCF-7 cells was investigated using fluorescent Annexin V-FITC/ PI detection kit (*BioVision* EZCell™ Cell Cycle Analysis Kit Catalog #K920) by flow cytometry assay. MCF-7 cells at a density of 2×10^5 per well were harvested and washed twice in PBS. After that, the cells were incubated at 37 °C and 5% CO_2. The medium was incubated with the tested compound **6b** at the IC_{50} (µM) for 48 h, washed twice in PBS, fixed with 70% ethanol, rinsed again with PBS. Afterward, medium was stained with DNA fluorochrome PI for 15 min at 37 °C. The samples were immediately analyzed using Facs Calibur flow cytometer (Becton and Dickinson, Heidelberg, Germany).

S4.2.4. Apoptosis assay for compound 6b

Apoptosis in MCF-7 cells was investigated using fluorescent Annexin V-FITC/ PI detection kit (*BioVision* Annexin V-FITC Apoptosis Detection Kit, Catalog #: K101) by flow cytometry assay. MCF-7 cells at a density of 2×10^5 per well were treated with compound **6b** at the IC_{50} (μM) for 48 h, then the cells were harvested and stained with Annexin V-FITC/ PI dye for 15 min in the dark at 37 °C. The samples were immediately analyzed using *FACS Calibur* flow cytometer (Becton and Dickinson, Heidelberg, Germany).
